# Climate and environmental change drives *Ixodes ricinus* geographical expansion at the northern range margin

**DOI:** 10.1186/1756-3305-7-11

**Published:** 2014-01-08

**Authors:** Solveig Jore, Sophie O Vanwambeke, Hildegunn Viljugrein, Ketil Isaksen, Anja B Kristoffersen, Zerai Woldehiwet, Bernt Johansen, Edgar Brun, Hege Brun-Hansen, Sebastian Westermann, Inger-Lise Larsen, Bjørnar Ytrehus, Merete Hofshagen

**Affiliations:** 1 Norwegian Veterinary Institute, Ullevålsveien 68, P.O.Box 750, Sentrum 0106, Oslo, Norway; 2 Georges Lemaître Centre for Earth and Climate Research, Earth & Life Institute, Université Catholique de Louvain, Place Louis Pasteur 3, B1348, Louvain-la-Neuve, Belgium; 3 Centre for Ecological and Evolutionary Synthesis (CEES), Department of Bioscience, University of Oslo, P.O.Box 1066, 0316 Blindern, Oslo, Norway; 4 The Norwegian Meteorological Institute, Research and Development Department, Division for Model and Climate Analysis, P.O.Box 43, 0313 Blindern, Oslo, Norway; 5 Department of Informatics, University of Oslo, P.O.Box 1080, Blindern 0316, Oslo, Norway; 6 Department of Infection Biology, Institute of Infection & Global Health, University of Liverpool, Leahurst Campus, Chester High Road, Neston, CH64 7TE Wirral, UK; 7 Northern Research Institute, P.O. Box 6434, Forskningsparken 9294, Tromsø, Norway; 8 Norwegian School of Veterinary Science, Ullevålsveien 72, P.O.Box 8146, Dep., 0033, Oslo, Norway; 9 Department of Geosciences, University of Oslo, P.O.Box 1066, Blindern 0316, Oslo, Norway

**Keywords:** Tick, Range expansion, Climate change, Climatic variability, *Ixodes ricinus*, *Anaplasma phagocytophilum*, Bush encroachment, Ecotones, Global environmental change, Remote sensing

## Abstract

**Background:**

Global environmental change is causing spatial and temporal shifts in the distribution of species and the associated diseases of humans, domesticated animals and wildlife. In the on-going debate on the influence of climate change on vectors and vector-borne diseases, there is a lack of a comprehensive interdisciplinary multi-factorial approach utilizing high quality spatial and temporal data.

**Methods:**

We explored biotic and abiotic factors associated with the latitudinal and altitudinal shifts in the distribution of *Ixodes ricinus* observed during the last three decades in Norway using antibodies against *Anaplasma phagocytophilum* in sheep as indicators for tick presence. Samples obtained from 2963 sheep from 90 farms in 3 ecologically different districts during 1978 – 2008 were analysed. We modelled the presence of antibodies against *A. phagocytophilum* to climatic-, environmental and demographic variables, and abundance of wild cervids and domestic animals, using mixed effect logistic regressions.

**Results:**

Significant predictors were large diurnal fluctuations in ground surface temperature, spring precipitation, duration of snow cover, abundance of red deer and farm animals and bush encroachment/ecotones. The length of the growth season, mean temperature and the abundance of roe deer were not significant in the model.

**Conclusions:**

Our results highlight the need to consider climatic variables year-round to disentangle important seasonal variation, climatic threshold changes, climate variability and to consider the broader environmental change, including abiotic and biotic factors. The results offer novel insight in how tick and tick-borne disease distribution might be modified by future climate and environmental change.

## Background

Ticks, currently the main vectors of arthropod-borne pathogens in Europe and a major threat to human and animal health [1], are increasing in abundance and expanding their distribution limits [2]. Identifying the multiple factors that may influence vector distribution is a prerequisite in predicting health risks for humans and animals. The limits of the range, as is found in Norway, are ideal grounds to unravel factors delineating population persistence or extinction. However, the factors and/or scale of changes at these extreme limits might not reflect the changes at the core distribution of the vector or pathogen [3].

*Ixodes ricinus*, a three-host tick species which is free-living with brief feeding periods between the different tick stages (larvae, nymph and adult) [4] transmits protozoal, viral and bacterial pathogens, several of which are zoonotic. As *I. ricinus* is ectothermic, its fitness is strongly temperature dependent [5], but its activity and survival are also closely related to the degree of relative humidity [6,7]. Global climate change is affecting overall mean temperatures and factors such as precipitation, rainfall, and vegetation, which in turn might affect the geographic distribution of ticks and other arthropods [2,8]. Beyond changes in the absolute levels of environmental variables, the probable changes in variability between seasons have also been highlighted [9,10]. Although these climatic changes could influence the life cycle of *I. ricinus*, we lack clear evidence for a consistent association between tick abundance and a warmer and wetter climate [11]. Other environmental variables such as landscape characteristics and abundance of hosts are also important drivers of tick population dynamics [12,13] and might therefore modify or mask climatic factor effects [14,15].

Landscape and hosts are also subject to climatic factors, as well as to agriculture, forestry and wildlife management practices [16]. Indeed, because of the multiplicity of interactions between factors influencing the tick abundance and dynamics, the effects of climate change on ticks and tick-borne diseases are often controversial and subject of debate [17]. A recent distribution map of *I. ricinus* in Norway [18] shows a shift in latitudinal and altitudinal distribution. During the last three decades there have been considerable changes in temperature, landscape, vegetation, demography, agricultural/forestry practice and the density of host animals in Norway.

The aim of the present interdisciplinary study was to explore the multi-factorial influence of biotic and abiotic factors in driving the expansion of *I. ricinus*. While spatially and temporally detailed tick distribution data is unavailable for the past, the prevailing distribution of *I. ricinus* in Norway corresponds to the distribution of *Anaplasma phagocytophilum*[19]. The bacterium *A. phagocytophilum* causes Tick-borne fever (TBF), which is the most common vector-borne pathogen of sheep and cattle in northern Europe [20]. Recent studies indicate that different strains of *A. phagocytophilum* may affect different host species and there may be variation in the pathogenicity of strains, even those affecting the same species of host [20]. *A. phagocytophilum* also causes human granulocytic anaplasmosis (HGA), which is now widely recognised as an emerging zoonotic tick-borne disease [21,22].

Changes in tick exposure can be indirectly measured by detecting infections or evidence of infection in hosts susceptible to tick-borne pathogens. As sheep are susceptible hosts to *A. phagocytophilum*[23], antibodies to *A. phagocytophilum* should be good indicators of the presence of *I. ricinus*, the only known vector of TBF in northern Europe [20]. Even though the prevalence of *A. phagocytophilum* in sheep also could be influenced by the rates of infection in the tick vector, density of hosts and the strains of *A. phagocytophilum* found within a geographic area, changes in the prevalence of *A. phagocytophilum* in sheep should reflect changes in tick exposure [24,25]. We studied changes in exposure to *I. ricinus* as proxied by the number of sheep seropositive for TBF.

All the explanatory variables were smoothed on a decadal basis to even out year to year variation. We focused on factors affecting survival and reproduction success of ticks, namely climate, bush encroachment, demography, abundance of cervids and farm animals.

## Methods

### Study design

Three study districts were selected in Southern Norway (INLAND, COAST and FJORD) which differ with respect to historical tick presence, topography, demography of human and animal population, bush encroachment, presence of cervids and degree of climate change. Sheep serum samples from farms residing in the three districts were divided into 3 timespans; timespan 1 (1978–1989), timespan 2 (1990–1999) and timespan 3 (2000–2008). Several environmental, climatic and demographic variables were generated for the same timespans as the serum samples, and their effect was tested using statistical models.

### Collection of samples and sample size

The serum samples belong to the sample and culture collection of the Norwegian Veterinary Institute (NVI), and were collected randomly, throughout the year, as part of the national surveillance programs (see Additional file 1). Each of 90 farms was sampled once (except for four farms which were sampled two (2), three (1) and four (1) times). A total of 2963 samples were collected. Assuming a seroprevalence of *A. phagocytophilum* of 10% in sheep flocks, we aimed for 300 samples per timespan within each district to be able to estimate prevalence with a 95% confidence limit and accuracy of 10% ± 3.39% (CI 0.069-0.140) using the R package Epi.

### Study area

Five municipalities were selected in the districts of Aust-Agder and Vest-Telemark (INLAND), an inland area previously thought to be free of *I. ricinus*[26,27]. Seven municipalities were selected in the district of Jæren (COAST), an exposed coastal and agricultural district with no or low abundance of ticks in the past [26,27]. Two municipalities were selected in the district of Haugalandet (FJORD), a more sheltered fjord and valley district, where *I. ricinus* has been common since the first surveys [26,27]. *I. ricinus* is now present in all municipalities that constitute these three districts [18]. A description of the landscape, vegetation and general climate in INLAND, COAST and FJORD is given in Additional file 1.

### Laboratory method

An enzyme-linked immunosorbent assay (ELISA) was used to test for the presence of antibodies against *A. phagocytophilum* in sheep [28,29], with minor modifications (Details given in Additional file 1). The absorbance value of each test sample was expressed as a ratio of positivity (PP). The cut-off point between positive and negative samples was 0.20 PP. This was based on the mean PP value + 2 standard deviations of several negative ovine sera [29].

### Definition of the outcome variable

The unit of observation was a single serum sample from an individual sheep. The outcome variable was the presence (≥ 0.20 PP) or absence (< 0.20 PP) of antibodies to *A. phagocytophilum* in each serum [29].

### Climatic data

As climatic variables tend to be spatially homogenous over short distances, each study district was divided in three zones depending on elevation and distance to the sea. Three representative farms were then chosen in each zone, resulting in 27 farms for which climatic variables were derived. Climatic variables were also generated for the rough grazing used by 22 farms at a different elevation or more than 10–20 km away from the farm. Further details are given in Additional file 1.

### Remote sensing data

Landsat images covering the 3 study districts were retrieved for the summer of either 1984 or 1988, and 2006 (Landsat 5TM). A binary map of bush encroachment from the 80’s to 2006 was produced. Buffer zones of a 500-m radius were established around farms and rough grazing locations. The total area of bush encroachment, the number of patches found within each zone, and their mean area were calculated (See Additional file 1). The bush encroachment variables were assumed to reflect a continuous process during the study period and were assumed to be independent of timespan.

### Demographic factors, grazing systems and the animal populations

The number of bagged cervids (moose, roe deer and red deer), number of sheep over one year of age, number of livestock farms and the human population in the municipalities were retrieved from Statistics Norway for 1980–2008. Changes in these factors during the study period are displayed in Additional file 1: Figures S2, S3 and S4 and Table S2.

The grazing system of each farm was identified on the basis of information given by the farmers and/or by the municipal agricultural offices. Two types of grazing systems were encountered: infield grazing in fenced pastures near or around the farm and rough grazing in semi-natural forest/mountain pastures away from the farm during the summer and autumn. The altitude ranges of the grazing areas are given in Additional file 1: Table S1. Fifty-eight farms (64%) grazed the sheep on mountain pasture whilst 32 farms (36%) kept the sheep on fenced pastures near the farm. All the farms in INLAND used mountain pastures at high elevation (mean 880 masl), whilst in COAST and FJORD 52% and 77% of the farms respectively used rough grazing, at a far lower altitude (mean 318 masl and mean 515 masl, respectively).

### Preprocessing of climate and temporal variables

Eleven of the twenty-two climatic variables for farm and pasture level were calculated on a monthly basis and the rest annually (Table 1). Highly correlated variables were identified using the first and second components of principal component analyses, and were subsequently grouped using means. For example, as the relative humidity variables for the winter months were highly correlated, they were grouped for the months October-March (RHmean_Oct-Mar_). The aggregated climatic variables were not highly correlated (all pairwise Pearson correlation coefficients < 0.7), and were assumed to account for the main seasonal patterns of the respective climatic variables. All temporal predictor variables were aggregated over a ten-year timespan matching the timespan defined for the outcome variable. For example, the moose population from the 1980’s (timespan 1), represent the mean bagged moose population during the period 1980–1989, 1990’s (timespan 2) defined as 1990–1999 and 2000’s (timespan 3) as 2000–2008. For the climatic variables the definition of the timespan period took into account tick biology. Ticks might complete the life cycle in three to six years. Therefore, climatic variables were aggregated for extended periods starting 4 years prior to the sampling timespans.

**Table 1:** Definition of all climate and temporal variables used in the analyses

**Variable for both infield and rough grazing level**	**Definition**
Moose	The number of bagged moose in the municipality (divided by size of municipality)
Red Deer	The number of bagged red deer in the municipality (divided by size of municipality)
Roe Deer	The number of bagged roe deer in the municipality (divided by size of municipality)
Sheep	The number of sheep in the municipality (divided by size of municipality)
NuFarms	The number of farms in the municipality (divided by size of municipality)
F_masl	The meters above sea level at which the farm is situated
Humans	The number of inhabitants in the municipality (divided by size of municipality)
Area	Denoting district 1,2 and 3 (INLAND,COAST and FJORD)
Timespan	Denoting the 3 decades; timespan 1(80s), timespan 2(90s) and timespan 3(00s)
Nu_patch	Number of patches of bush encroachment in a 500-m radius
Meanarea_p	Mean area of patches of bush encroachment intersected by a 500-m radius (m^2^)
Area shrubi	Total area covered by patches of bush encroachment intersected by a 500-m radius (m^2^)
TMeanJan; TMeanFeb; etc.….	Daily mean air temperature; monthly basis
TMeanSDJan; TMeanSDFeb; etc…	Daily mean air temperature standard deviation; monthly basis
TminJan; TminFeb; etc..	Lowest daily mean air temperature; monthly basis
TmaxJan; TmaxFeb; etc..	Highest daily mean air temperature; monthly basis
GrowSeasDays	The length of the growing season. **(1)**
RRSumJan; RRSumFeb; etc..	Precipitation sum, monthly basis
RH > 70DaysJan; RH > 70DaysFeb; etc..	Number of days with relative humidity >70%; monthly basis
RHMeanJan; RHMeanFeb; etc..	Mean relative humidity; monthly basis
SatDefMeanJan.; SatDefMeanFeb; etc..	Mean saturation deficit; monthly basis. **(2)**
SatDef < 5DaysJan; SatDef < 5DaysFeb; etc..	Number of days with saturation deficit <5 mmHg; monthly basis
SnoStartDays	Number of days in a hydrological year* to snow depth ≥2 cm **(3)**
SnoEndDays	Number of days in a hydrological year* to snow depth ΓΫ ≤2 cm in spring
SnoDepth ≥ 2Days	Number of days in a hydrological year* with snow depth ≥2 cm
SnoDepth1-2Days	Number of days in a hydrological year* with snow depth of 1–2 cm
SnoDepth2-20Days	Number of days in a hydrological year* with snow depth of 2–20 cm
SnoDepth > 20Days	Number of days in a hydrological year* with snow depth >20 cm
SnoSum	Sum of snow depth (cumulative) per hydrological year*
GSTminJan	Lowest daily ground surface temperature (GST); monthly basis
GSTmaxJan	Highest daily ground surface temperature (GST); monthly basis
FTDays-SnoDepth ≥ 2	Number of days in a hydrological year* with freeze-thaw events at ground surface with snow depth ≥ 2 cm. **(4)**
FTDays-SnoDepth < 2	Number of days in a hydrological year* with freeze-thaw events at ground surface with no snow cover or snow depth <2 cm.
BlackFrdays	Number of days in a hydrological year* with black frost; daily GST < 0°C and ground bare of snow or snow depth < 2 cm.
TDecr÷5 < DaysJan; TDecr÷5 < DaysFeb; etc..	Number of days per month where temperature decrease in GST from a day to the next day are >5°C.
TDecr÷10 < DayJan; TDecr÷10 < DayFeb; etc..	Number of days per month where temperature decrease in GST from a day to the next day are >10°C.
TIncr + 5 < DaysJan; TIncr + 5 < DaysFeb; etc..	Number of days per month where temperature increase in GST from a day to the next day are >5°C.
TIncr + 10 < DaysJan; TIncr + 10 < DaysFeb; etc..	Number of days per month where temperature increase in GST from a day to the next day are >10°C.

**(1)** Number of days between the end of the first continous 4-day period with a 24 h mean air temperature > 5°C and the beginning of the last continous 4-day period with a 24 h mean air temperature >5°C. **(2)** Saturation Deficit (SDF) is a measure of air humidity (in mmHg); it is the difference between actual and maximum vapour content: SDF = (1 − RH/100) × 4.9463 × e(0.0621T). RH is the daily mean RH (%), T is the daily mean air temperature (°C). **(3)** For all SnoDays variables and SnoSum the number of days refers to the hydrological year. **(4)** A freeze-thaw event is defined as when daily Ground Surface Temperature (GST) crosses 0°C from one day to the next. *A hydrological year is from 1 September – 31 August.

### Statistical analyses

The prevalence data at individual sheep level (presence or absence) were fitted with a mixed effect logistic regression. In order to account for spatial and temporal structure in the data, the variables “municipality” and “timespan” were designated as random effects in the mixed effect logistic regression. Four municipalities were combined with a neighbouring municipality such that each random effect level contained data from at least two farms. The multivariable regression was performed with the function lmer (lme4 package in R) and family binomial. All analyses were performed in R version 2.14.0 [30]. Generalized additive models [31] were used to identify possible nonlinear relationships or skewed variable distributions. These exploratory analyses suggested second-order polynomial terms for four variables, and categorisation of a skewed variable.

We used a two-step procedure for model selection. Initially we fitted single variable mixed effect logistic regressions of all potential predictor variables with municipality and timespan as random effects and kept only variables with a p-value ≤ 0.20 for further model selection. Twenty-eight of 67 variables were kept for the farm/infield and 18 of 67 variables for the rough grazing (see Additional file 1: Table S3). A stepwise backward model selection approach was applied to build the full model, using Akaike Information Criteria (AIC) for model selection [32]. Highly correlated variables (Pearson correlation coefficient ≥ 0.7), were not included in the full model at the same time. A difference of ≤ ± 2 of the AIC value was regarded as equivalent models and the most parsimonious model was then chosen. Residuals of the final model were plotted against all explanatory variables, and mapped, in order to explore any potential remaining systematic patterns. The predictive power of the final model, with random effects included, was assessed by plotting predicted farm prevalence against observed farm prevalence. To measure the accuracy of the model, area under the curve (AUC) of a ROC curve was calculated. The ROC curve is a plot of the sensitivity versus (1- specificity) for all thresholds [33].

## Results

### Antibodies against *Anaplasma phagocytophilum*

**Figure 1:**
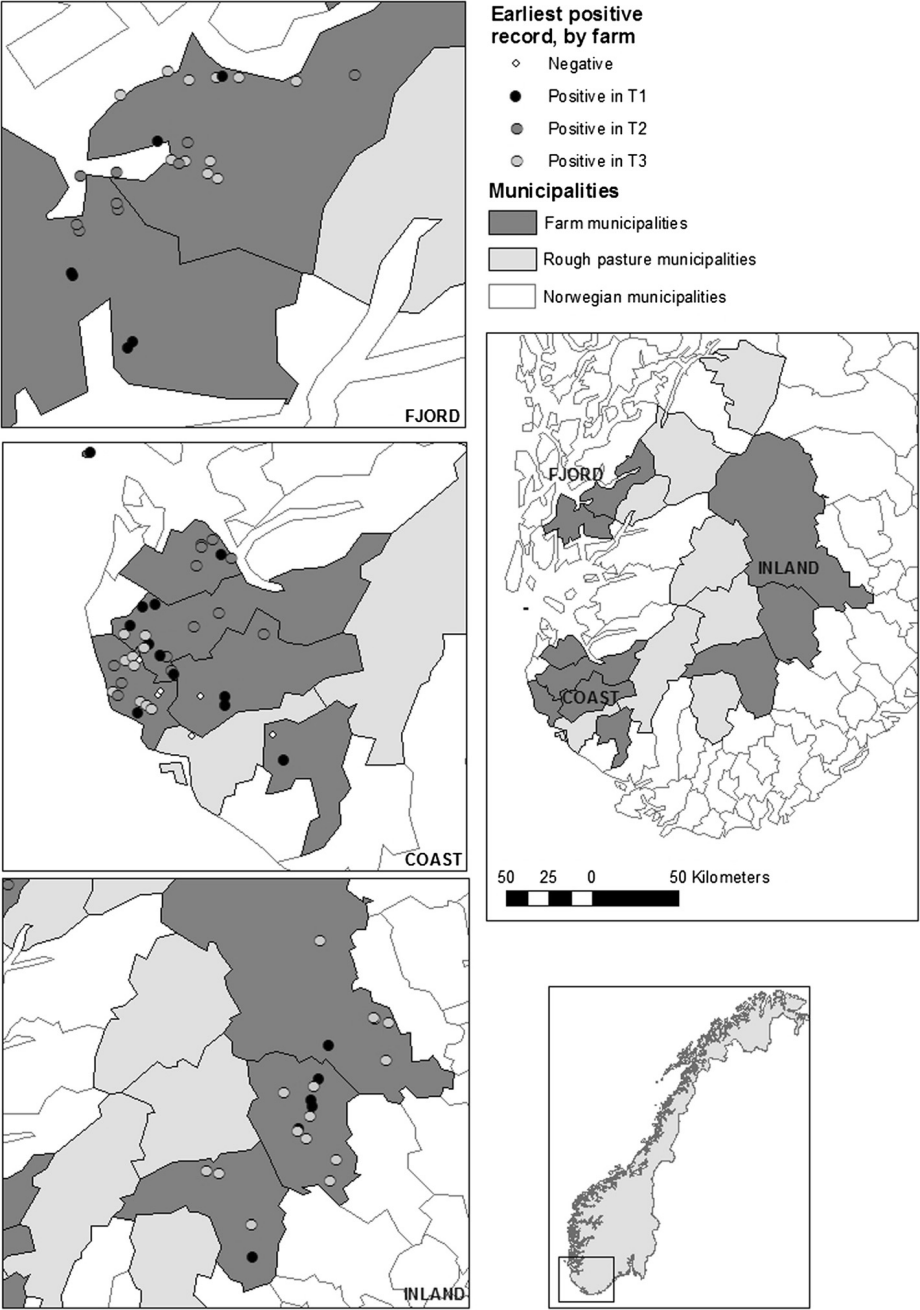
**Geographic distribution of farms which were positive and negative for antibodies against *A. phagocytophilum *during timespan 1, timespan 2 and timespan 3 in the three regions (INLAND, COAST and FJORD).** A positive farm is defined as a farm with one or more positive samples. A negative farm has no positive samples.

A total of 1543 samples (52%) were positive for antibodies against *A. phagocytophilum* (Figure 1). Seropositive sheep were detected in all areas and during all timespans (Additional file 1: Table S1 and Table S2). There was an increase in the number of positive animals over time for FJORD, an increase for the last time span in COAST whilst there was a decrease over time in positive samples collected from INLAND (Table 2). FJORD showed the highest number of positive animals (Table 2).

**Table 2:** **Changes in the prevalence of antibodies against *A. phagocytophilum *** **in sheep and farms**

**District**	**Timespan**	**Farms tested (n)**	**Sheep tested (n)**	**Positive sheep (n)**	Proportion positive sheep [95%CI]*	**Mean farm prevalence [90% range]**
INLAND	1	6	379	139	0.37 [0.32-0.42]	0.33 [0.18 – 0.46]
	2	0	0	n.a	n.a	n.a
	3	16	436	124	0.28 [0.24-0.33]	0.26 [0.06 – 0.52]
COAST	1	12	520	252	0.48 [0.44-0.53]	0.59 [0.34 – 0.92]
	2	20	403	117	0.29 [0.26-0.35]	0.39 [0.00 – 1.00]
	3	12	339	210	0.62 [0.57-0.67]	0.61 [0.13 – 1.00]
FJORD	1	6	240	151	0.63 [0.56-0.69]	0.72 [0.14 – 1.00]
	2	9	326	249	0.76 [0.71-0.81]	0.78 [0.47 – 1.00]
	3	11	320	301	0.94 [0.91-0.96]	0.94 [0.80 – 1.00]

In the 3 study regions during three different timespans: timespan 1; 1978–1989, timespan 2; 1990 – 1999; timespan 3: 2000–2008. *Confidence intervals obtained from assuming that the positive sheep are independent and following a binomial probability distribution.

### Climatic variables

**Figure 2:**
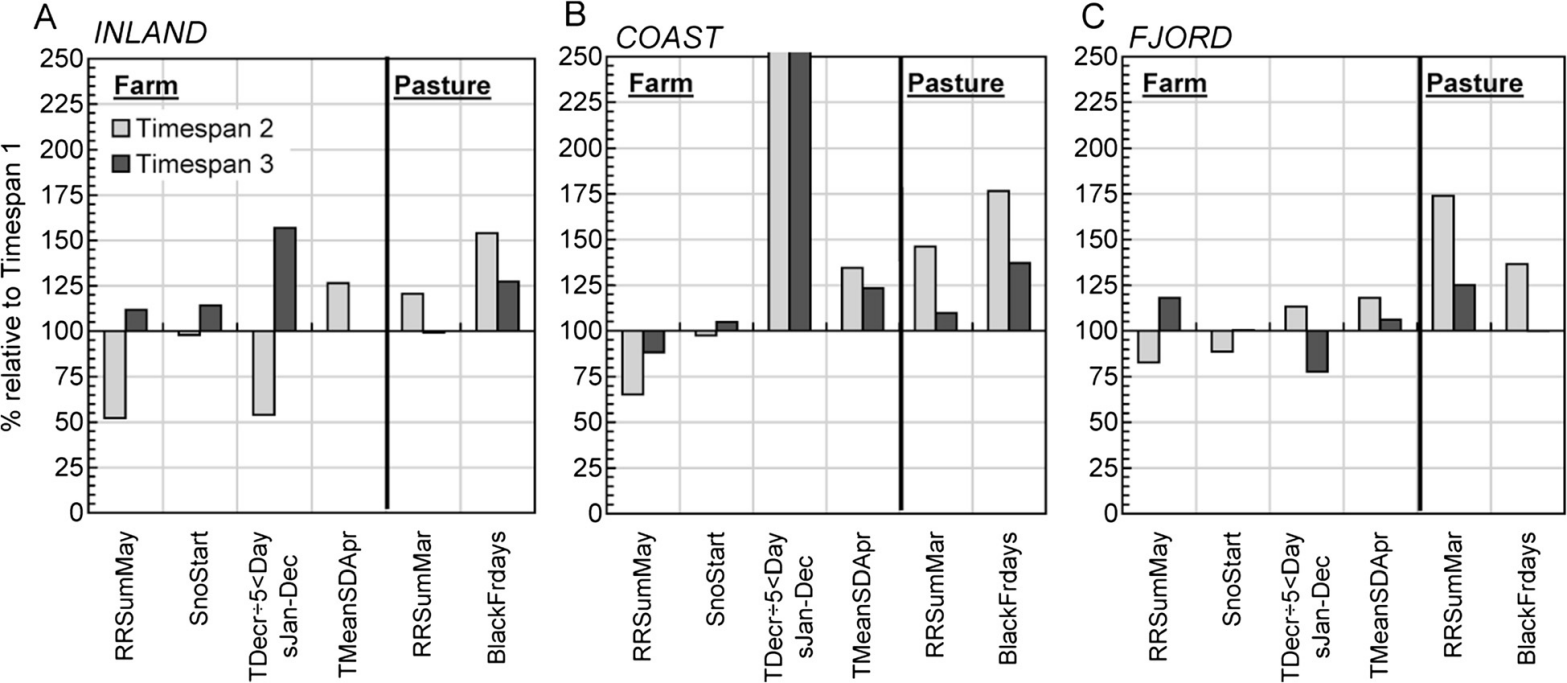
**Changes over time in the specific climate variables, which was significantly associated with the outcome in the multivariable model. (A)** INLAND, **(B)** COAST and **(C)** FJORD. Farm denotes the variables significant at farm level, whilst pasture denotes variables significant at rough grazing level. Changes are shown for timespan 2 (1990-1999) and timespan 3 (2000-2008) relative (in %) to timespan 1 (1980-1989). RRSum_May_ : Precipitation in May; SnoStartDays: Number of days from 1 September - 31 August to snow depth ≥2 cm, TDecr_÷5<_Days_Jan-Dec_ :  Number of days per month where temperature decrease in Ground Surface Temperature (GST) from a day to the next day are >5 °C; RRSum_Mar_ : Precipitation in March; and BlackFrdays: Number of days from 1 September – 31 August with black frost, daily GST < 0°C and ground bare of snow or now depth < 2 cm.

The general trends in essential climatic variables for period 1981–2010 are described in Additional file 1: Figure S1. Figure 2 displays some variables significant in the multivariable model (Table 3). INLAND is characterized by higher inter-annual and decadal climate variability and shows more variations between the timespans. COAST had the most prominent and homogenous changes. The greatest increase was in the number of days per year with high day-to-day fluctuations in ground surface temperature (TDecr÷5 < Days_Jan-Dec_, definitions in Table 1). FJORD had the least prominent changes over time compared to INLAND/COAST, but still at a level of > 20% change for several variables.

**Table 3:** **The output (parameter estimates, standard errors and p-values) of the mixed effect logistic regression (see Table **1**for definitions)**

**Variable**	**Estimate**	**Std. error**	**Exp(est)**	**P-value**	**Δ AIC**
Intercept	2.30	0.84		0.006	
Area shrubi 1 vs. 0*	1.05	0.27	2.86	<0.001	14
Area shrubi 2 vs. 0*	0.85	0.23	2.35	<0.001	
Area shrubi 3 vs. 0*	0.79	0.23	2.21	<0.001	
Meanarea_ p	0.45	0.12	1.40	<0.001	9
Meanarea _p^2^	−0.11	0.03		<0.001	
RHmean_*Oct-Mar*_	1.21	0.13	3.34	<0.001	88
BlackFrdays	0.92	0.16	2.50	<0.001	55
SnoStartDays	1.17	0.20	3.23	<0.001	34
NuFarms	2.67	0.31	2.27	<0.001	94
NuFarms^2^	−1.85	0.21		<0.001	
Red deer	1.28	0.22	3.59	<0.001	29
Pasture	−1.36	0.30	0.25	<0.001	18
RRSum_*May*_	−0.18	0.15	0.59	0.224	46
$\mathrm{RRSu}m_{\mathrm{May}}^{2}$	−0.35	0.06		<0.001	
RRSum_*Mar*_	−0.40	0.14	0.67	0.004	6
TIncr + 5 < Days_*Jun*_	−1.11	0.21	0.40	<0.001	27
$\mathrm{TIncr}+5<\mathrm{Day}s_{\mathrm{Jun}}^{2}$	0.19	0.04		<0.001	
TMeanSD_*Apr*_	0.43	0.22	1.93	0.047	5
$\mathrm{TMeanS}D_{\mathrm{Apr}}^{2}$	0.22	0.08		0.004	
TDecr ÷ 5 < Days_*Jan* − *Dec*_	0.22	0.10	1.25	0.035	2

Δ AIC denotes the change in AIC level obtained if excluding the relevant variable from the selected model. The continuous variables are scaled (before taking polynomials) to mean zero and variance one. The factor by which the odds of positive outcome are increased for each one-unit change in the variables are represented by the computed exp (estimates). For the polynomials the odds ratio is calculated only for an increase of one standard deviation from mean. ICC (Intraclass correlation; ratio of the variance between subjects over the total variance) for municipality was 0.33 and ICC for timespan was 0.36. The “Area shrubi” variable was categorized (in 4 equal parts defined by quartiles) to capture the nonlinear relationship (at logit-scale) with the outcome. The variables Area shrubi, BlackFrDays and RRSumMar represent the rough grazing level.

### Vegetation changes

Bush encroachment was observed in all study areas. INLAND had the most pronounced bush encroachment of farm surroundings and rough grazing. On average 10 patches of bush encroachments intersected a 500 m radius around the farm, totalling on average about 5ha (6.4% of the buffer area). On the other hand, 500 m radii around rough grazing locations intersected on average 8 patches of bush encroachment, totalling an average 3.5 ha (4.5%). INLAND also had the largest mean size of encroached patches, with 0.5 ha near farms and 0.3 ha in rough grazing locations. COAST, a more intensively used region where agricultural land covers most of the area, had on average 2 patches of bush encroachment intersecting a 500 m radius, totalling less than 1ha (1.3%) on average in both farm and pasture locations. COAST also had the smallest average mean size of patches, below 0.2 ha both around farms and in pastures. FJORD presented an intermediate situation, with 4 and 6 patches intersected on average in farms and on rough grazing respectively, totalling just over 1ha (1.3%) around farms and just over 2 ha (2.5%) in rough grazing locations.

### Results for multivariable regression

**Figure 3:**
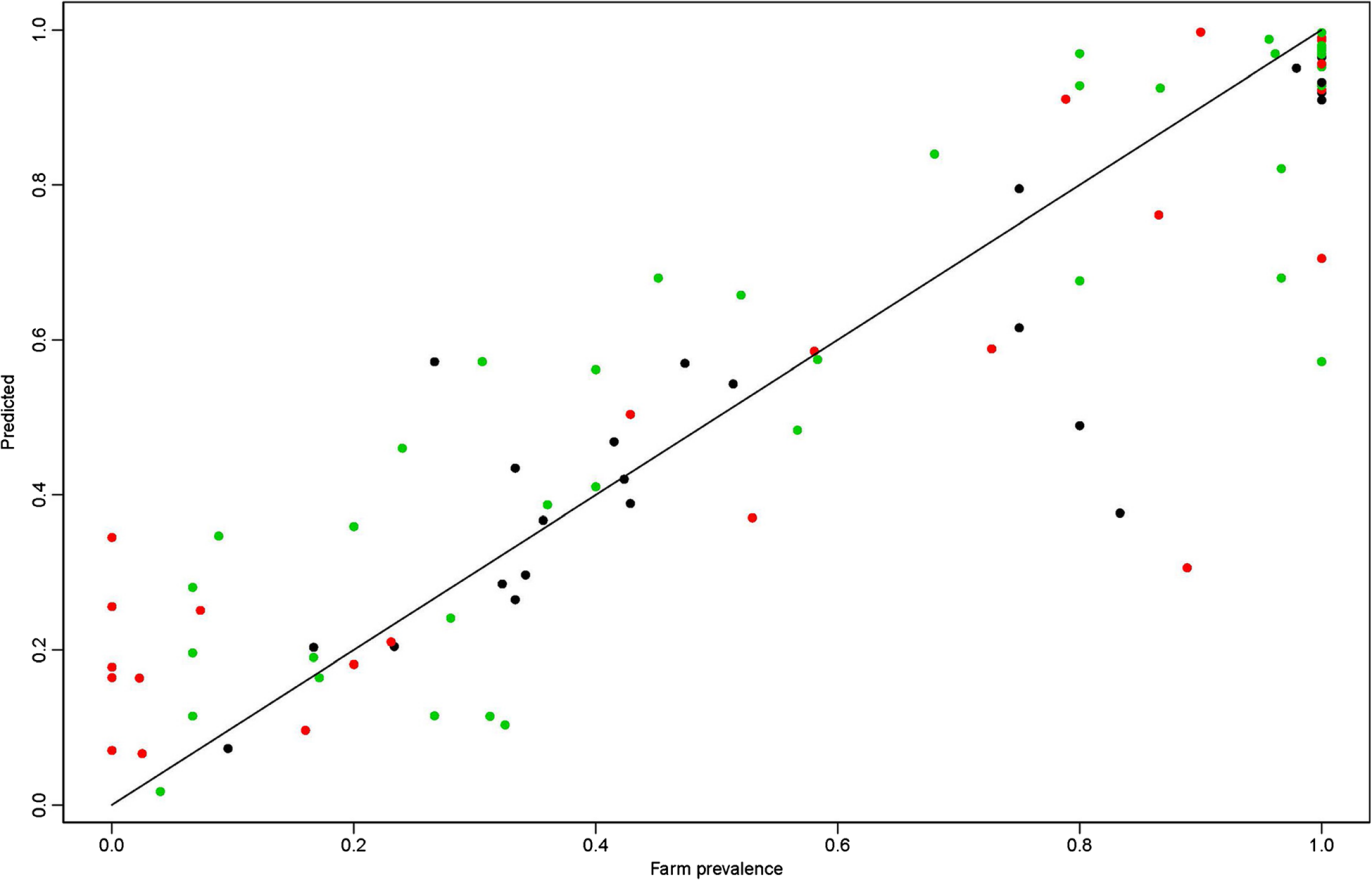
**Prediction of the presence of *A. phagocytophilum *at farm level using the final model.** The colour black denotes timespan 1, red timespan 2 and green timespan 3. The black line is the regression line of observed versus predicted presence with an intercept of −0.013 and a slope of 1.023 which give an adjusted R2 of 0.80.

The model that best predicted *A. phagocytophilum-*prevalence in the three districts over time included large daily fluctuations in temperature in general, and in certain months of the year, seasonal precipitation changes, duration of snow cover, bush encroachment, abundance of red deer and density of farms with animals (Table 3). The six variables explaining most of the variation (as assessed by the AIC value), were the density of farms, relative humidity from October–March, yearly number of days with black frost, precipitations in May, duration of snow cover and abundance of red deer. The model predicts the observed farm prevalence very well (Figure 3). The AUC of the resulting model was 0.85.

### At farm/infield

The mean patch area of bush encroachment, density of livestock farms, abundance of red deer, number of days per year with day-to-day fluctuation in ground surface temperature (GST) >5°C, number of days with snow depth of ≤ 2 cm (SnoStartDays), standard deviation of mean air temperature in April and relative humidity during October-March were positively associated with the outcome. Of these variables, the highest impacts on the probability of a positive outcome were found by one unit increase in red deer abundance, SnoStartDays, and relative humidity during October–March (Table 3). The numbers of days in June with day-to-day increase in GST of >5°C was negatively associated with the outcome. The amount of precipitation in May had a positive significant association with increased prevalence of TBF in sheep at lower values, and a negative relationship at higher values (> 90 mm). An increase by 1 standard deviation from the mean amount of May precipitation (from 93 mm to 113 mm) decreased the probability of a positive outcome by 0.6 (95% CI: 0.4, 0.9).

### At rough grazing

The total area of bush encroachment and the number of days with black frost during the year were positively associated with the outcome. The probability of a positive outcome was approximately 2.5 times higher for the three bush encroachment categories with more bush encroachment than the lowest category defined by the first quartile of the variable “Area shrubi”. An increase by one unit in the black frost variable increased the probability of a positive outcome by 2.5 times (95% CI: 1.8, 3.4). The amount of precipitation in March was negatively associated with the outcome.

## Discussion

Understanding the response of populations to environmental change requires detailed spatial and temporal data, and also consideration of the variability in conditions and of non-climatic factors along with the climate [34,35]. Many disease distribution models use means, ignoring fluctuations, and will probably not be able to predict the diversity of animal responses. Incorporation of non-climatic factors, such as biotic interactions, is especially important [36,37]. Our models covered climate and its variability, habitat alteration, and changes in host animal populations, agricultural practices and demography through three decades.

### Distribution of *Ixodes ricinus*

The upslope shift of *I. ricinus* is even greater than documented previously [18], with a positive INLAND farm at 748 masl. Sheep serum positive for *A. phagocytophilum* in INLAND and parts of the COAST districts (Figure 1) in the early 1980’s did not correspond with published distribution maps [26,27]. This might be caused by different data resolution [18].

FJORD had higher numbers of positive sheep compared to COAST, probably indicating that ticks were relatively more abundant in FJORD. The number of positive sheep increased in FJORD, and more recently in COAST, indicating an increased sheep-tick exposure over time. It might also indicate that as *A. phagocytophilum*, which causes persistent infections in sheep [38] gets established in an area, there will be more reservoir hosts for more ticks to be infected and spread the disease. It is more difficult to explain why in INLAND the number of positive sheep decreased over time. One possible explanation is that a reduction in the number of sheep farms in INLAND might have resulted in less contact between herds during summertime, possibly lowering the infection risk for sheep coming from areas where ticks are uncommon. In addition, climate change was less pronounced and the INLAND-farms/rough grazing were at higher altitudes, however, INLAND had the most pronounced degree of bush encroachment. The prevalence of *A. phagocytophilum* in sheep could also be influenced by the tick infection prevalence rate, the tick life cycle and the density of sheep, cattle and wildlife hosts. The specific strains of *A. phagocytophilum* circulating might also be of importance as some may not be infective to sheep.

As whole cell bacterial antigens were used, it is expected that the method will detect antibodies against all strains of *A. phagocytophilum* that infect sheep. However, as not all strains of *A. phagocytophilum* infect sheep, the results could underestimate the distribution of ticks. Using prevalence of *A. phagocytophilum* in sheep as a proxy to establish temporal changes in the range and density of ticks could potentially miss tick populations which do not harbour *A. phagocytophilum* or strains of the organism that do not infect sheep, and thus underestimate the range and density of tick populations.

One possible weakness of the present study is that it did not investigate the prevalence of *A. phagocytophilum* in *I. ricinus* during the study period. There is, however, a problem of inference since prevalence in questing and feeding ticks can differ vastly; e.g. a Belgian study found only an *A. phagocytophilum* prevalence of 3% in questing ticks, whilst a prevalence of 21.7% in feeding ticks [39]. Thus low prevalence in questing ticks might not be indicative of infection rates.

### Climatic factors

#### Climate variability – tick population and A. phagocytophilum transmission

The data indicates that there was a significant increase in the frequency of monthly drops in temperature with time in COAST and INLAND during the latter timespan (Figure 2). Climate change may increase temperature variability [8]. Raffel *et al*. [33] concluded that an increase in the severity of unpredictable drops in temperature might be relevant to disease transmission. Ectotherms generally adapt to temperature variations by a left-skewed asymmetric response [40] resulting in a non-linear relationship between body temperature and fitness. In addition, temperate ectotherms, like those living in Norway, have a lower optimal temperature relative to the species maximum temperature, and display a greater asymmetry compared to Mediterranean and tropical ectotherms. Furthermore, studies on the malaria vector (*Anopheles stephensi*) and pathogen (*Plasmodium chabaudi*) have revealed that fluctuations around the lower temperatures (16-18°C) speed up intensity of pathogen transmission, whilst fluctuations around higher temperatures (24-26°C) might slow it down [41]. Such fluctuations probably also affect the stress resistance of the vector, and the contraction and multiplication of the infectious agents within the vector [41,42]. Paaijmans *et al*. [41] have shown that the mosquito and parasite are influenced by the extent of daily temperature variations and suggest the need to consider this for other ectotherms. This might explain why large fluctuations in temperature were positively associated with the presence of *Anaplasma-*infected ticks. In a theoretical model, temperature variability was found to strongly affect the time at which *I. ricinus* cohorts emerged [43].

#### Duration of snow cover – tick population

Climatic changes during the last 30–40 years in Southern Norway have had the clearest impact in winter [44]. Warmer winters with less snow and increase in spring temperatures are accompanied by earlier spring snow melt and bare ground, which causes rapid spring warming and greater drying of soils and vegetation [45]. The absence/presence of snow cover, rather than the length of the growth season, was significant for tick distribution. As long as the snow is absent ticks can still quest, given that the temperature during some period of the day is high enough, even though the growth season has ended. The prominent coastal distribution of *I. ricinus* in Norway is better explained by the length of the period without snow than the length of the growth period. James *et al*. [46] also found that the length of the growth season was not significant when modeling environmental determinants of *I. ricinus* in Scotland. However, a study on the distribution of *I. ricinus* in Sweden regards the length of the growth season as the best predictor for tick distribution, but this study also described *I. ricinus* as consistently present when the period of snow cover was ≤ 125 d/year and as consistently absent with a snow cover ≥ 175 d/year [47]. The importance of relative humidity during the winter months probably results from the impact on ticks that are not protected by snow cover. Snow cover affects the survival of ticks during winter [48], as it increases ground surface temperatures [49] and ensures stable and high relative humidity in the air. In places with little or no snow the ticks are not protected, and the absence of snow together with low relative humidity will probably kill the ticks. The significance of number of days with black frost in the rough grazing areas might reflect that absence of snow cover during the winter is far more crucial for *I. ricinus* than the actual exposure temperature. Precipitation in March serves as an indicator of the duration of snow cover in INLAND and areas of higher elevation in COAST and FJORD, as the precipitation consists mostly of snow. An accumulation of snow can delay the start of spring in the rough grazing areas, rendering the habitats less favourable for ticks and their hosts.

#### Spring temperature – tick population

Increased temperature fluctuations during April (Figure 2), are often linked to the presence of persisting high-pressure systems producing sunny, calm weather with gradually increasing air temperatures. April is normally the month with the highest degree of temperature rise, and marks the transition from winter to spring. The IPCC reports that there is very high confidence that there is an earlier start to spring [50], and since 1900 the temperature increase in Norway has been greatest in spring [44]. An early exit from diapause can be critical for interstadial development and thus the presence and abundance of ticks [51]. Dobson *et al*. [51] suggested that developmental diapause exit for *I. ricinus* is around 1st of April in England – and that April-July is a critical phase for the tick inter-stadial development. Additionally, the effects of climate conditions on winter/spring survival of important tick hosts need to be considered, as was observed for white-footed mice (*Peromyscus leucopus*), in Canada, an important tick larval host [52]. It is possible that persisting high-pressure systems also have an indirect effect through increased survival of small animal hosts for the ticks.

#### Precipitation in May – tick population

The effect of precipitation in May (Figure 2) had a nonlinear effect. There was a positive relationship with low precipitation values, and a negative one for high precipitations (> 90 mm per month). Since the 1990s, precipitations have increased substantially in May [44] (Figure 2). A recent study by Dyrrdal *et al*. [53] showed that the frequency of moderate to strong precipitation events has increased in most parts of Norway since 1957, particularly in wet regions. The intensity of strong precipitation events has also showed a general increase. IPCC reports that climate change very likely has increased the frequency of observed heavy precipitation events [50].

Studies on *I. scapularis* from Ontario in Canada [54] and *I. ricinus* at several British sites [51,55], climatically similar to some places in Norway, indicate that egg deposition take place during late April - May. The climatic factors during the month of egg deposition will probably be critical for the distribution of ticks, as there would not be sustainable tick population if the eggs do not survive and hatch. The effect of flooding upon the dog ticks (*Rhipicephalus sanguineus* and *Haemaphysalis leachi leachi*) shows that flooding affected oviposition, reduced the number of eggs laid and the percentage of hatchability [56]. It has been suggested that heavy rain silts up the egg masses [57]. Other studies have shown that light-to-moderate rainfall is favourable for oviposition, whilst excessive rainfall reduces oviposition and tick distribution [58,59].

Firm interpretation of significant climate variables is challenging, but these results highlight the role of variability and directions for future research on the role of climate.

#### Land cover and bush encroachment- tick population and A. phagocytophilum transmission

In this study we focused on one specific aspect of vegetation change, bush encroachment. This change may be related to a more favorable environment for ticks [60-62], especially in areas where it connects to other landscape types [60,63]. Trees and bushes probably provide a more stable and humid microhabitat, thereby potentially enhancing tick survival [60]. At farm- level, beyond the direct effects of increasing areas more suitable for ticks and some hosts, the size of patch would relate to the presence of ecotones, that is, interfaces between bushy or woody vegetation and grasslands, with more possibilities for interaction between suitable areas and susceptible sheep.

Woodlands are the natural habitat for *I. ricinus*[63] as they provide a favorable environment and a diversity of hosts. Small mammals and wild cervids circulate between woodlands, ecotones and pasture areas and this movement of animals affects the distribution of *I. ricinus*[64]. In rough grazing, the exact area used by sheep is impossible to outline, and the characterization of those areas may be more indicative, resulting in more generic variables, such as area, being significant. Bush encroachment of open fields in Norway is linked to decreasing numbers of farms, changed forestry practices, change of agricultural use and climate change [65]. Forest regrowth is likely to ultimately replace bush in encroached areas. Regrowth after abandonment or reduced use and climate change are concurrent, and hard to separate. At this stage, land use might have a greater impact on forest regrowth than climate change in Norway [65,66]. Rough grazing is considered the main restricting factor for forest re-growth [67]. In Sweden and Latvia, an increase in the prevalence of tick-borne disease has been associated with abandonment of fields and pastures and with the expansion of woodland [68,69].

#### Domestic animal hosts - tick population and A. phagocytophilum transmission

The significance of the number of livestock farms in the municipality could be related to the fact that the presence of more farms will provide more ruminants and thus major tick hosts. This would be in contrast to the widespread belief that reintroduction of sheep and cattle to an area will alleviate the tick burden through the effect of grazing on the vegetation [70,71]. However, densities in Norway may be insufficient to affect tick habitat suitability as was observed elsewhere. A higher number of farms might be associated with increased contact between herds on rough grazing. Herds from tick-infested areas will then seed ticks into the rough grazing pasture areas, possibly causing increased tick exposure for other herds utilizing the same areas. Anthropogenic activities such as farming have also the potential to change the availability and density of hosts and vectors, and thus indirectly influence the spread and persistence of infectious pathogens within an ecosystem [72-74].

#### Cervid populations - tick population and A. phagocytophilum transmission

In the multivariable analyses red deer was the only cervid that had a significantly positive association with the outcome. Roe deer and moose were not associated with the outcome. The role of these species might be different depending on the *A. phagocytophilum* strain.

The strains infecting roe deer belongs to a different *ank*A gene cluster than those infecting red deer, sheep, European bison and cows, and roe deer might not be relevant reservoirs for granulocytic anaplasmosis in humans and domestic animals [75]. Red deer has been reported as hosts for 16S rDNA variants of *A. phagocytophilum,* known to cause TBF in sheep [76]. This might provide an explanation as to why red deer, and not roe deer, were significant. However, msp4 genotyping performed indicated a clustering of wild ruminant strains distinct from sheep variants [76]. There are several distinct *A. phagocytophilum* 16S rDNA and msp4 gene variants circulating in sheep and wildlife in Norway [77], six 16S rDNA and 24 msp4 variants have been reported [76,77]. The transmission cycle of *A. phagocytophilum* in Europe is not completely understood and we do not know the specific role of the different tick host species in the transmission cycle [75]. Infection risk for *A. phagocytophilum* over the years might thus potentially be related to changes in one of the reservoir species.

Cervid species are regarded as key hosts to ticks [78,79]. High population densities of cervids are expected to be associated with efficient host finding and adequate nutrition for ticks. However, cervids may also affect tick abundance through the effect of grazing on vegetation. Results from studies on the influence of roe deer on tick abundance are conflicting [80-85] even though positive associations between roe deer and tick abundance have been reported [86-88]. Evidence points towards a far more complex relationship between deer and tick density, where for instance density of deer above threshold values might have little effect on tick abundance [89,90] or a decoupling of stage-specific tick abundances can occur [81]. The relationship between deer abundance and tick birth rate is probably difficult to predict because ticks can aggregate on fewer deer or alternate hosts in response to abundance declines.

Hunting data at the municipality level was used as a proxy for the total population of cervids. The number of bagged cervids is reported to reflect the overall size of the total population [91], although some urge caution in using wildlife bag data since differences in spatio-temporal patterns between bag data and population size have been detected [92].

## Conclusion

Tick distribution and tick bite exposure are associated with a complex combination of climatic and environmental factors, including those related to human activities, which operate at diverse spatial and temporal scale. This multifactorial interdisciplinary study contributes to a more comprehensive understanding of the intricacies and interactions of the drivers of shifts in *I. ricinus* distribution, and represents an advance by considering biotic and abiotic factors simultaneously. The study also integrates seasonality, short-term temperature dynamics and possible climatic threshold effects, which appeared essential even though interpretation remains challenging. Expected climate changes accentuate the importance of our finding, and the need for considering climate variability effects upon ticks and tick-borne pathogens. The relative importance of the different factors studied here might change as the global environment continues to change, including the respective role of abiotic/biotic factors and those related to human land use.

## Competing interests

The authors declare that they have no competing interests.

## Authors’ contributions

The study was planned in collaboration between the authors with SJ as a project leader. SJ did the majority of manuscript writing, but all co-authors contributed with improvements and discussions. SJ, HV and ABK carried out the data analysis. All authors read and approved the final version of the manuscript.

## Supplementary Material

Additional file 1 Supplementary material. Click here for file
