# Supplementary material for: Climate and environmental change drives Ixodes ricinus geographical expansion at the northern range margin
Source: Parasit Vectors. 2014 Jan 8;7:11. doi: 10.1186/1756-3305-7-11 (PMC3895670; doi:10.1186/1756-3305-7-11)
Supplement: Additional file 1 — Supplementary material. [file 1756-3305-7-11-S1.docx]

**Climate and environmental change drives *Ixodes ricinus* geographical expansion at the northern range margin**

**Additional file 1**

S. Jore^1^*, S.O. Vanwambeke^2^, H. Viljugrein^1,3^, K. Isaksen^4^, A.B. Kristoffersen^1,5^, Z. Woldehiwet^6^, B. Johansen^7^, E. Brun^1^, H. Brun-Hansen^8^, S. Westermann^9^, I.L. Larsen^1^, B. Ytrehus^1^ and M. Hofshagen^1^

^1^Norwegian Veterinary Institute, Ullevålsveien 68, P.O.Box 750 Sentrum 0106 Oslo, Norway

^2^Georges Lemaître Centre for Earth and Climate Research, Earth & Life Institute, Université Catholique de Louvain, Place Louis Pasteur 3, B1348 Louvain-la-Neuve, Belgium

^3^Centre for ecological and evolutionary synthesis (CEES), Department of Biology, University of Oslo, P.O.Box 1047, Blindern, 0316 Oslo, Norway

^4^The Norwegian Meteorological Institute, Research and Development Department, Division for Model and Climate Analysis, P.O.Box 43 Blindern, 0313 Oslo, Norway,

^5^Department of Informatics, University of Oslo, P.O.Box 1080, Blindern, 0316 Oslo, Norway

^6^Department of Infection Biology, Institute of Infection & Global Health, University of Liverpool, Leahurst Campus, Chester High Road, Neston, Wirral CH64 7TE, United Kingdom

^7^Northern Research Institute, P.o.b 6434 Forskningsparken, 9294 Tromsø, Norway

^8^Norwegian School of Veterinary Science, Ullevålsveien 72, P.O.Box 8146 Dep., 0033 Oslo, Norway

^9^Department of Geosciences, University of Oslo, , P.O.Box 1066, Blindern, 0316 Oslo,

**Corresponding author*:

[Solveig.jore@vetinst.no](mailto:Solveig.jore@vetinst.no)

**Contents**

[Additional file 1 Introduction 3](#_Toc350506798)

[Additional file 1 Methods 3](#_Toc350506799)

[Additional file 1 Discussion](#_Toc350506804) 12

[Additional file 1 References 27](#_Toc350506804)

# Additional file 1 Introduction

In this supplementary material we present data and methodology in more detail.

# Additional file 1 Methods

**Description of the study area**

The sampling frame consisted of three geographical areas: five interior municipalities of the county of Agder and Telemark (INLAND): Kviteseid, Fyresdal, Tokke, Vinje og Bygland, seven municipalities belonging to the county of Rogaland (COAST): Sandnes, Lund, Bjerkreim, Hå, Time, Gjesdal and Kvitsøy and two municipalities in of Ryfylke, northern part of Rogaland county and Hordaland county (FJORD): Vindafjord and Etne. According to published tick distribution maps [1-3] we would not expect to have ticks present in the 80’s in the selected municipalities of Agder and Telemark, but expect to see some changes during the decades. The treeless and wind-swept Jæren, was according to Tambs-Lyche, free from ticks. Lastly, in the county of Rogaland ticks have been present for a long time, but changes in seroprevalence might have occurred through the decades. Samples were selected from the three different regions from the 80s, the 90s and from year 2000 and onwards.

The elevation of farms in INLAND (mean 377 meters above sea level (masl)), was higher than those of the COAST (mean 109 masl) and FJORD (mean 57 masl). Two types of grazing systems were encountered: infield grazing in fenced pastures near or around the farm and rough grazing in semi-natural forest/ mountain pastures away from the farm during the summer and autumn. On average infield pastures were located at 169 masl (range 10 – 748 masl) and the rough grazing pastures varied between lower bounds (range: 136-1400 masl and mean: 448.7 masl) and at higher bounds (range: 173.8-1530 masl and mean: 658.6 masl).

INLAND is characterized by deep valleys with small patches of agricultural land along rivers and deep, long inland fjords. Many valley sides are covered by dense spruce forest, with pine at higher altitudes, and also large areas of bare rock. Hills and mountains separating the valleys are covered with forests with scattered pine and birch trees and large areas of peat bogs. Mountain tops are low and rounded with thin soils only partly covered with grass, lichens, mountain birch and crowberries.

The outer, western part of COAST consists of a relatively flat agricultural landscape dominated by fields, meadows and cultivated pastures divided by old stone walls. There are relatively few large trees, though small hedges and plantations of coniferous trees appeared over the last century. The eastern part of the district is a hillier landscape consisting of small valleys with creeks and lakes surrounded by agricultural land. The valleys are interrupted by rocks, cliffs and outcrops almost without soils. The vegetation is low, mostly dominated by heather, but in sheltered spots small forests of birch and oak may grow.

FJORD consists of a rugged, but lush and moist landscape of richly branching fjords and valleys. The valley bottoms often have nutritious soils and are characterized by small fields, meadows and pastures divided by temperate broadleaf forests with oak, maples, birches and other deciduous trees. At higher altitudes, forests are dominated by pine, with occasional spruce plantations. The valleys and fjords are divided by small, rugged and rounded mountains with much bare rock, only partly covered with blueberries and mountain grasses

The INLAND municipalities are partially lee areas in relation to the large weather systems mainly coming from the west. However the westerly municipalities receive more precipitation than the easterly area. There is also a gradient from the coast to the inland, which results in the driest areas in the north-easterly parts of the municipalities. In spring the mountainous areas in the north of Aust-Agder and Telemark have a mean air temperature between −6 and −2 °C. During the summer the warmest areas are in the south-east with mean air temperature of 14-16 °C. The areas in the west are under the influence of the North Sea and have lower summer mean air temperature, i.e between 12 and 14 °C. The lowest mean air summer temperature is found in the mountainous areas with around 6 °C at the highest elevations. The region can during the summer months experience considerable precipitation with thunderstorms which often is connected with periods of high air temperatures. During the autumn and winter the distance to coast is most important for determine the air temperature. Along the coast the mean air temperature is above 8 °C while mean temperature in mountainous areas is close to 0 °C. During winter the mean air temperature along the coast is between 0 and 2 °C, while negative mean air temperatures dominate the rest of the region.
COAST: There are two primary drivers of the weather – the sea and the high mountains further into the countryside. This leads to a mild and humid climate. The frontal precipitation dominates, and most of the precipitation is received during autumn and winter. The driest areas (e.g. Kvitsøy) have an annual precipitation of about 1100 mm. The wettest areas have more than 2000 mm. In the springtime the mean air temperature is around 4-6 °C and during the summer 13-15 °C and the area also receive a lot of precipitation. During the autumn Jæren belongs to the hottest areas on the west coast, the mean air temperature is around 8 °C. During the wintertime the mean air temperature is 0 °C and the precipitation is primarily consisting of rain. FJORD: this area has many mutual weather characteristics with Jæren. However due to the nearby mountains both frontal and orographic precipitation dominates, thus receive more snow and considerably more precipitation compared to the outermost areas of Jæren.

**Description of the development of the cervid population in the study area**

The most prevalent cervid in INLAND is the moose. Since 1980, the number of hunted moose showed a steady increase until around 1999 when there was a moderate decline in the growth (0.2 shot moose/ km^2^) followed by a moderate decline starting in 2005. Red deer was virtually absent from INLAND in the 1980s and only few animals were shot in the 1990s, but from the start of the 2000s this species became more prevalent in the area with 147 animals shot in 2007 (0.04/ km^2^). The roe deer population in this relatively snow-covered and cold area is typically relatively small and characterized by rapid fluctuations largely influenced by the winter conditions.

Back in the 1980s the most prevalent cervid in COAST used to be roe deer. The population in this area showed a steady increase through the study period, from 0.025 animals shot per square kilometer in 1980 to 0.20 in 2008. However, while being absent from the area in the early 1980s, the red deer population of COAST started to increase from the early 1990s, equaling the roe deer hunting bag in 2005 and reaching 0.22 animals/km^2^ in 2007. The moose is only rarely seen in the western part of COAST, but there are persisting populations in the mountain pasture municipalities. The population has showed the same trends as the moose population in INLAND, with an increase up to the end of the 1990s followed by a moderate decline, but at a much lower density (0.04 animals/km^2^).

FJORD has been known as a red deer area for a long period of time. However, also in this area there has been a pronounced increase in hunting bags through the study period, increasing from 0.04 animals shot per km^2^ in 1980 to 0.27 in 2007.  In the 1980s roe deer was absent from the area, but has become more prevalent and in 2008 0.05 animals were shot per sqkm. Moose are rare in this area.

Overall, each district is dominated by a different cervid but all three areas have experienced a marked increase in abundance of cervids. However, the overall cervid density seems to vary between the areas, with INLAND having a much lower average density of cervids (0.2 shot cervids/ km^2^) compared to COAST and FJORD (0.38 and 0.28/ km^2^ respectively in 2005).

**Collection of samples and sample size**

The serum samples belong to NVI’s sample and culture collection. In the late 70s, 80s and 90s diagnostic samples were taken from sheep farms or contact farms to investigate serological evidence of infection with maedi-visna virus, Border disease virus or Louping ill virus (descriptive study; unpublished, J. Krogsrud). In 1997 a control programme was launched for meadi-visna in all flocks in high-risk regions (Rogaland and Hordaland counties) which lasted for seven years. From 2003 a nationwide surveillance and control program for maedi-visna was established by randomly selecting flocks of participating in ram circles were and testing all flocks belonging to the same ram circle. The collection of serum samples was carried out by official veterinarians. We aimed to get at least 300 samples from each decade for the chosen geographical area. The samples included were collected throughout the year. Negative controls for the laboratory analyses were sampled and tested in June 2010 from a farm located between 70-71°N.

**ELISA**

An enzyme-linked immunosorbent assay (ELISA) was used to test for the presence of antibodies against *A. phagocytophilum* in sheep, [4,5]with minor modifications.

Purified preparations of bacteria grown in tick cells, treated with 0.1% P-40 (Sigma), were used to coat ELISA microtitre plates by overnight incubation at 4^o^C in carbonate bicarbonate buffer (pH 9.6), 96-well flat-bottomed plates (MaiSorp Immunoplates, Nunc, Denmark) were coated with 50 μl of antigen diluted in carbonate bicarbonate buffer, pH 9.6, to give a final concentration of 20 μg/ml; the plates were then incubated overnight at 4ºC. The remaining binding sites were then blocked by adding 100 μl of 0.2% bovine serum albumin (BSA) in sample diluent (0.5M Tris-HCl, pH 7.4 and 1mM EDTA) and incubation for an hour at 37°C. The plates were washed five times with wash buffer. The test and standard positive and negative sera were diluted 1:200 in sample diluent buffer and 50 μl of each dilution added to triplicate wells. After incubation overnight at 4ºC, the plates were washed five times before adding 50 μl of an optimal dilution (in sample diluent) of monoclonal mouse anti-sheep IgG horseradish peroxidase (HRP, Sigma Aldrich, Poole, Dorset, UK). After incubation at 37ºC for 1 h, the plates were washed 5 times. Then 100 μl of freshly prepared soluble substrate for HRP (o-phenylenediamine dihydrochloride, as SIGMAFAST OPD, Sigma Aldrich, Poole, Dorset, UK), with optimal concentrations of fresh H_2_O_2_ were added to each well. The plates were left at room temperature in the dark for 20 min for colour development before stopping the reaction by adding 50 μl of 2.5N H_2_SO_4_. The optical density (OD) of each well was then determined using a micro-plate reader (MRX Microplate Reader, Dynex Technologies, Worthing, West Sussex, UK) with a test wavelength of 490 nm. Each test run included a positive reference serum and a negative control serum. The absorbance value of each test sample was then expressed as a ratio of positivity (PP) using the formula:

PP = $\frac{ODtest-ODnegative control}{\begin{aligned} ODpositive control-ODnegative control \\ \end{aligned}}$

OD: optical density

Positive control: serum from sheep experimentally infected with *A. phagocytophilum* and shown to have antibodies against *A. phagocytophilum*.

Negative control: serum from sheep bred and raised in tick-free environment and known to be negative for antibodies against *A. phagocytophilum*

The cut-off point between positive and negative samples was 0.20 PP. This was based on the mean PP value + 2 standard deviations of several negative ovine sera [5].

**Climatic data**

Air temperature and precipitation obtained from daily observations were interpolated to a 1x1 km^2^ grid covering the Norwegian mainland [6-8]. Daily grids have been made available since 1957 and can be accessed at www.seNorge.no. Air temperature was estimated from a residual kriging approach using terrain and geographic position to describe the deterministic component [9]. Precipitation was interpolated using triangular irregular networks (TINs). A terrain adjustment was performed on the precipitation grid, according to the assumption that precipitation increases by 10% per 100 m up to 1000 masl and 5% above that [10,11]. Precipitation and temperature grids are input in a precipitation/degree-day snow model with a snow routine similar to the HBV hydrology model (Hydrologiska Byråns Vattenbalansavdelning modell; Bergström, 1992) as described previously [12]. Temperature dependent thresholds were used to separate snow from rain (T = 0.5 °C) and to determine snow melt and refreezing (T = 0.0 °C). Snow depth was estimated from the amount of existing snow and fresh snow reduced by melting and compaction [13].

Relative humidity was obtained from a new set of a high-resolution hindcast data produced at The Norwegian Meteorological Institute [14,15]. It was produced using a hydrostatic numerical weather prediction model, the High Resolution Limited Area Model (HIRLAM) [16] with 10 km horizontal resolution and 40 vertical layers. The boundary values were taken from a global reanalysis project, ERA40, at the European Centre for Medium Range Weather Forecasts (ECMWF). After August 2002 the boundary values are from the operational weather forecasting model at ECMWF.

Ground surface temperatures were calculated using a soil thermal model, which numerically solves Fourier’s Law of heat transfer in the ground and snow cover [17]. The model accounts for latent heat effects during soil freezing and thawing and allows for a dynamic upper boundary, so that the build-up and ablation of the seasonal snow cover can be included [17]. The modeled soil domain extends to a depth of 100m, with a grid spacing increasing from 0.05m in the snow cover and in the close to the soil surface to 10m at the lower boundary. For each of the grid cells, the volumetric fractions of air, mineral and water are prescribed, with water changing to ice according to a freezing characteristic following Dall’Amico et al. 2011 [18]. Hereby the sum of water and ice contents remains constant in time. The thermal properties of each grid cell were calculated from the volumetric fractions of the soil constituents [17]. The model is driven by gridded data of air temperature and snow depth from the gridded data set described above. At the lower boundary a constant geothermal heat flux is prescribed. The soil domain is initialized to steady-state conditions of the first five years in the gridded data set and the soil thermal model is subsequently run at daily resolution. As ground surface temperature, we employ the temperature of the uppermost cell of the soil domain extending from the soil surface to a depth of 0.05m.

**Remote sensing data from Landsat 5**

Material

Landsat images were retrieved for the summer of 2006 (Landsat 5TM) and the summer of

1984/1988 (Landsat 5TM). All the Landsat TM/ETM+ data sets collected were geo-referenced to the UTM map format, zone 32, WGS84, using the control-point correction method with a root-mean square error of less than one pixel. The NDVI image processing was performed using ENVI image processing software. The geographic information analyses were performed on Arc Gis - Geographical Information system.

Various mathematical combinations of spectral channels have been applied as sensitive indicators of the presence and condition of green vegetation [19]. Most simple of the vegetation indices is the vegetation index (VI), defined as "the ratio between the near-infrared channel and the red channel". This equation has further been developed into the Normalised Difference Vegetation Index (NDVI). NDVI was found [19-21] to be a representative of plant assimilation condition and of its photosynthetic efficiency. NDVI is an indicator of the density of chlorophyll and leaf tissue calculated from the red and near infrared bands:

NDVI = (NIR-RED) / (NIR+RED)

In this equation NIR represents the Near Infrared band 4 (0.76-0.90 µm) of Landsat 5 and 7 and RED the corresponding band 3 (0.63-0.69 µm). NDVI gives values between -1 and + 1. Vegetated areas in general yield high values for these indices due to their high near infrared reflectance and low visible reflectance. Reflectance of cloud, snow and water is larger in the red than near infrared. Clouds and snowfields yield negative values while water has very low or slightly negative values. Rock and bare soil have approximately similar reflectance values in the red and near infrared channels, and results in indices near zero. A zero or close to zero means no vegetation. [22,23]. The NDVI is further used for deducing temporal changes in the vegetation cover. Temporal changes in NDVI are related to the seasonal changes in the amount of photosynthetic tissues; typically NDVI increases in spring, saturates at a certain point of greenness in summer and then declines in autumn, at mid to high latitudes. The NDVI equation has a simple, open loop structure. This renders the NDVI susceptible to large sources of error and uncertainty over variable atmospheric and soil background conditions, wetness, imaging geometry, and with changes within the canopy itself [21,22].

**Methods**

All images were processed into NDVI and mosaicked by time period. Images from the two time periods were then differenced to obtain the change in NDVI. Based on visual interpretation of aerial pictures from “Norway in pictures” (<http://www.norgeibilder.no/>), the NDVI difference image was sliced into 1) areas of significant change and 2) others. The binary map showing these two categories was overlaid with the CORINE map. Pixels that had important changes in NDVI, and which were classified as forest or semi-natural areas (broad-leaved forest, coniferous forest, mixed forest and transitional woodland shrub) in the 2006 CORINE map, were extracted for further analyses. These pixels were interpreted to constitute areas of bush encroachment, i.e. areas that in the last, but not the first time period had low woody vegetation. This resulted in a binary map of bush encroachment. Buffer zones of a 500-m radius were established around farms and pasture areas. The total area of bush encroachment within each zone was calculated. The structure of the bush encroachment was characterized by the number of patches found within each zone, and their mean area. No data was calculated for zones found in cloudy areas of the image, as per visual examination of the original images.

# Additional file 1 Discussion

***Results for multivariable regression***

The AIC for the null-model with outcome and only random effects (Timespan and municipality), was 3293, whilst AIC for the best model was 2748. The residuals of the final model showed no remaining pattern (for spatial pattern, see Additional file 1 Figure S6). All pairwise correlations between explanatory variables included and those not included in the final model were equal or less than 0.7, except for timespan with roe deer (0.73), red deer with district (0.76) and snow start with mean ambient temperature (0.74).

***Distribution of* Ixodes ricinus**

Even though *I. ricinus* is the only known vector of *A. phagocytophilum* in Northern Europe, other tick species could in theory transmit this pathogen. Of the few tick species present in Norway [1], *I. trianguliceps* would be the most plausible candidate. However, as it is very unlikely that any of the stages of this tick species feed on large mammals as sheep [24] this can be excluded. A cross-reaction between *A. marginale* and *A. phagocytophilum* antibodies has been reported [25]. However, as far as we know *A. marginale* infection has never been diagnosed in Norway.

***Climatic factors***

Annual air temperatures (Figure 1 panel A) have increased by 0.8-1.3 °C in all three districts, with INLAND having the greatest increase. Annual precipitation trends were not so clear due to several wet years in the early 80s (Figure 1 panel B). However, a positive increase of 5-10 % was recorded in several COAST and westernmost FJORD municipalities and southern and northern INLAND municipalities. In some of the easternmost FJORD and COAST and central INLAND municipalities precipitation trends were slightly negative. Trends in annual maximum snow depth (Figure 1 panel C) were generally negative in all three districts, except in the highest mountain and inland areas. This is in line with recent warming (cf. Figure 1 panel A), as more and more precipitation falls as rain and melting increases in lowland areas while maximum snow depth may increase during sufficiently cold winters [26]. The number of near-zero events (Figure 1 panel D, defined as days having daily mean temperature between −1.5 and 1.0 °C) had increased with time in INLAND and in the eastern pasture municipalities, while there were no clear trends in the COAST and INLAND farm municipalities. A and B are consistent with previous studies [27]. The results of the trend analysis are highly sensitive to the analysed time period and its length, due to the strong natural variability in Norway [26].

Uncertainties associated with the climate data mainly relate to the gridding procedure. The set of measurements used in the interpolation varies from day to day depending on availability and quality, which might have influenced temporal trends. According to Tveito et al. [8], temperature grids perform well except in cases of temperature inversion during winter time. Cross validation showed that the temperature grids generally perform better during the summer and that errors increase with decreasing temperatures. Precipitation is a more challenging element to interpolate because of its complex nature. Thus precipitation grids deviate more from reality. Despite the above-mentioned uncertainties we consider the data to be sufficiently accurate for the purpose of regional scale analysis.

**Figure S1**: Trends in relevant climate variables for period 1981-2010. Annual air temperature (A), Annual precipitation (B), Annual maximum snow depth (C) and Number of near-zero events (D). neg.: negative; pos.: positive; sign.: significant; notsign.: not significant. Statistical significance is at the 5% error level (C and D generated from Dyrrdal et al. [26]).


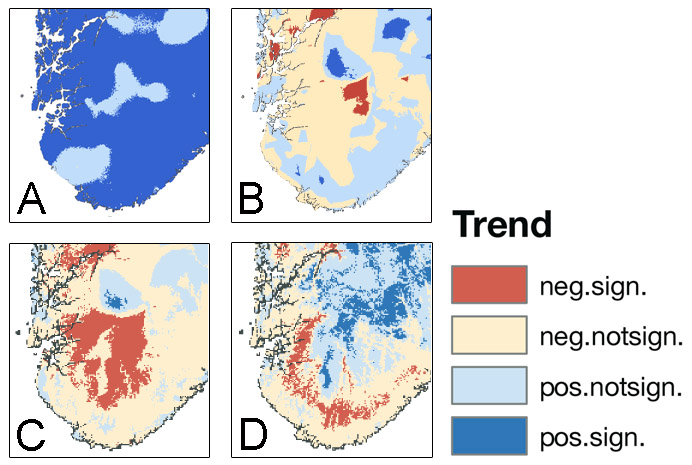


**Figure S2**: Abundance of Moose, red deer and roe deer during timespan 1 (1980’s) as

**Figure S2**: Abundance of Moose, red deer and roe deer during timespan 1 (1980’s) as represented by hunting bags recorded in the different districts during that period.


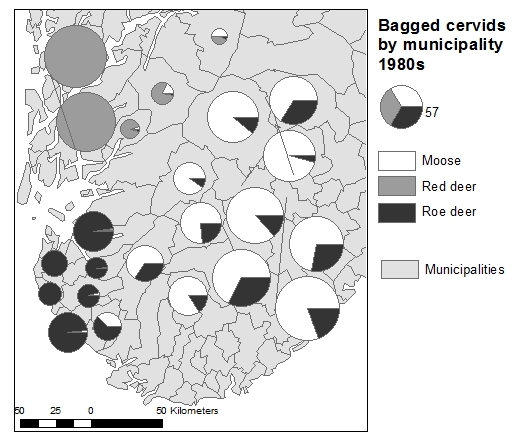


**Figure S3**: Abundance of moose, red deer and roe deer during timespan 2 (1990’s) as represented by hunting bags recorded in the differenct districts during that period. **
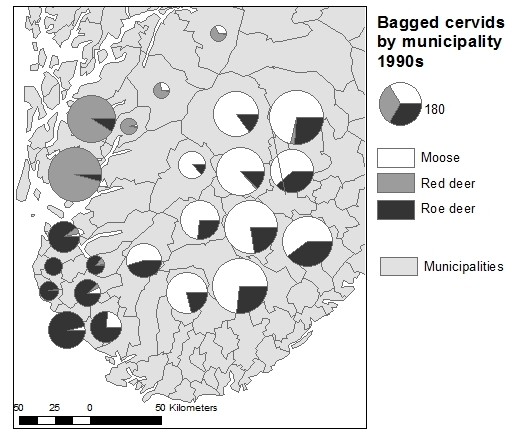
**

**Figure S4:** Abundance of moose, red deer and roe deer during timespan 3 (2000’s) as represented by hunting bags recorded in the differenct districts during that period.

**
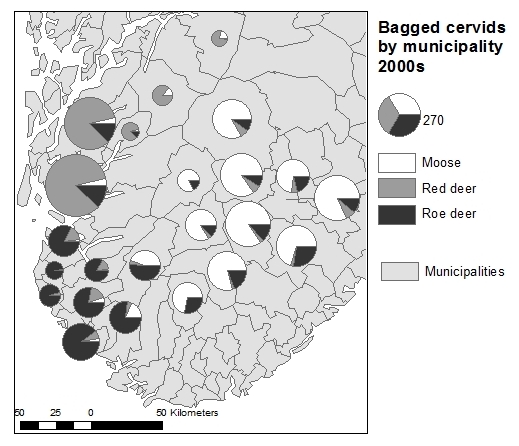
**


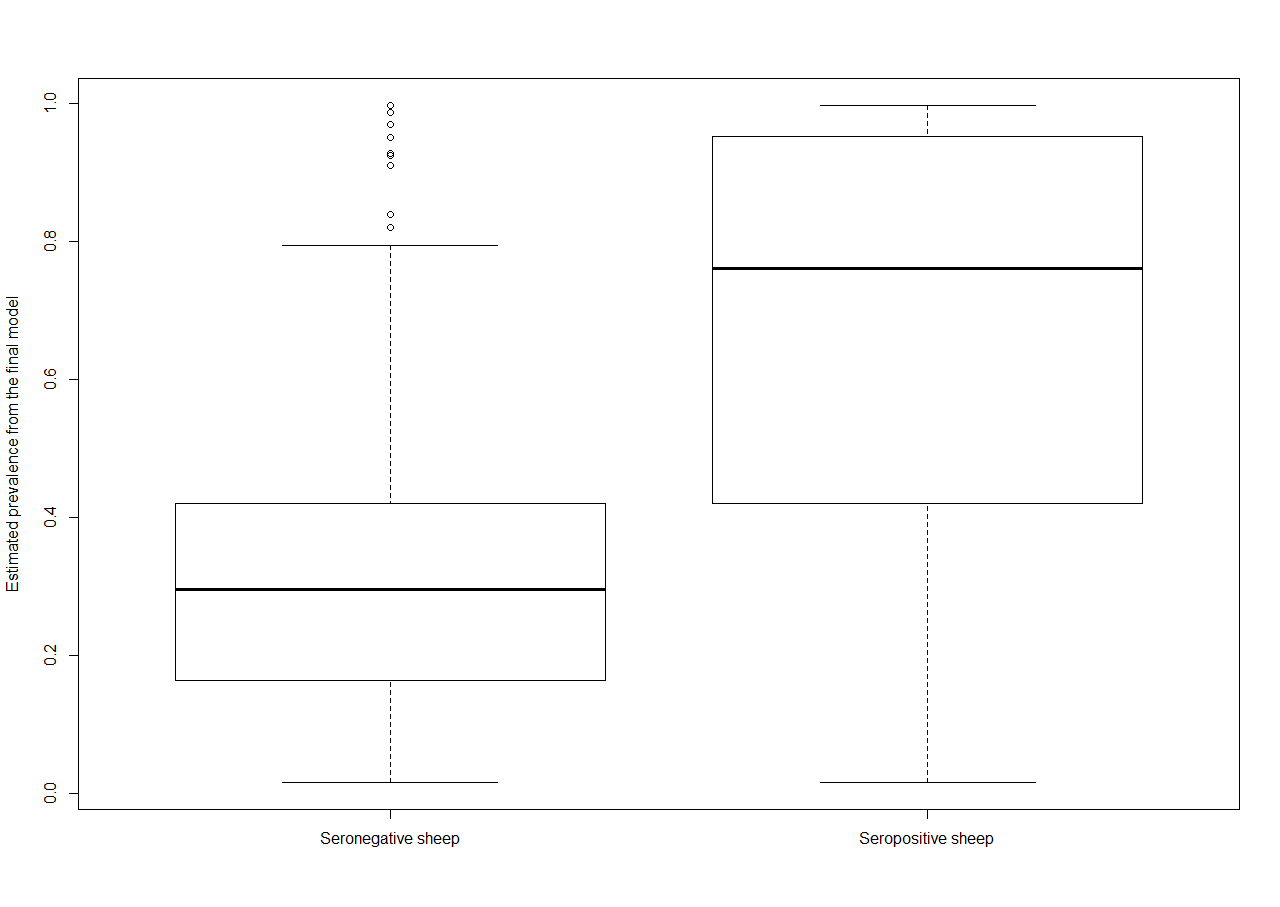
**Figure S5**: Estimated prevalence of seropositive and seronegative sheep in all districts using the final model.

**Figure S6**: Spatial plot of residuals after removing five outliers from the final model.


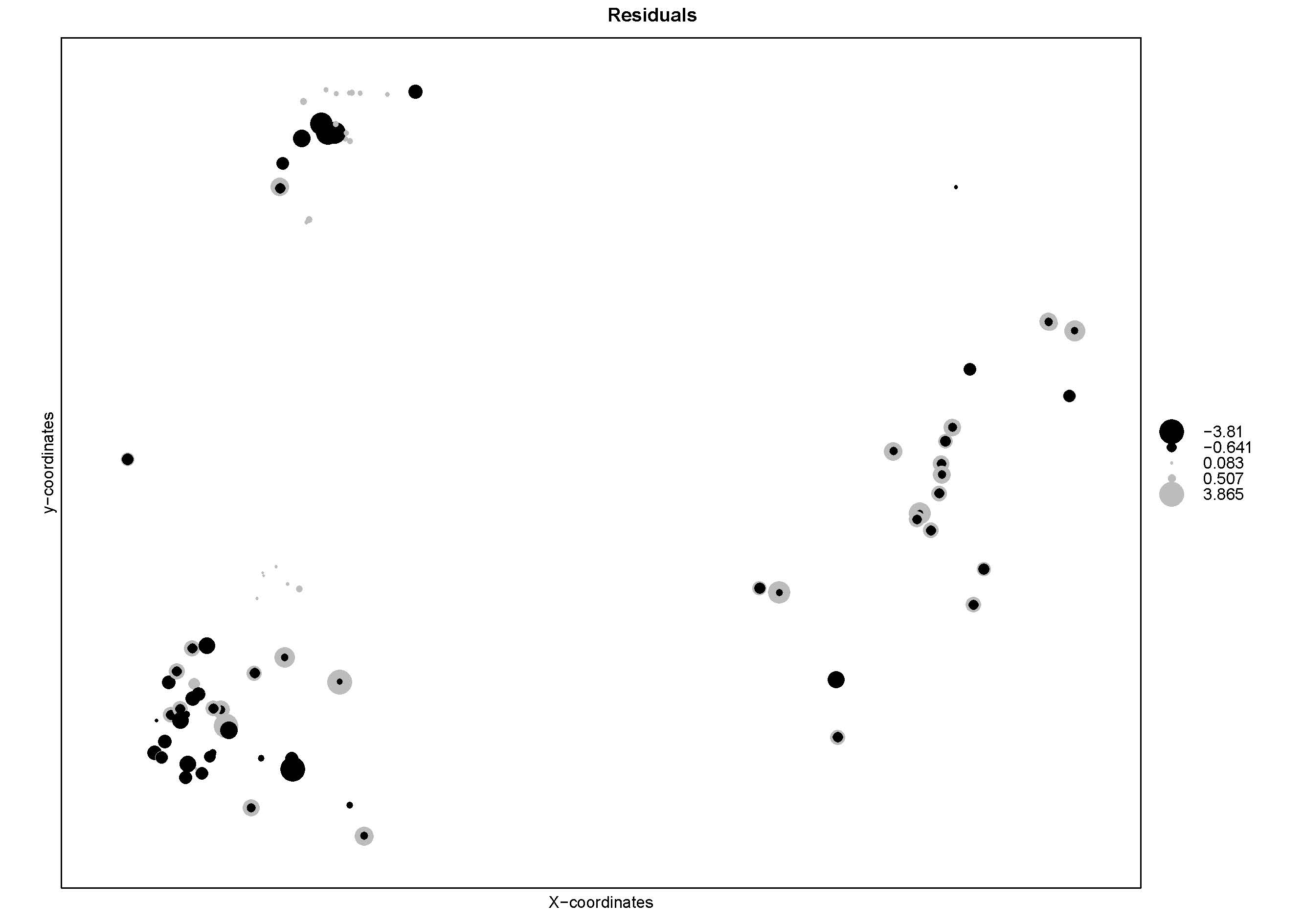
**Table S1:** Prevalence of *Anaplasma phagocytophilum* in farms in the three different geographical areas test during timespan 1 (1978 -1989), timespan 2 (1990 –1999) and timespan 3 (2000- 2008). *Four farms were sampled more than once; two of these were sampled during two different timespans.** This farm is one of the farms in Norway located at the highest altitudes. The farmer had not bought any ewes from other flocks but has used rams from nearby farms. Both the farmer and the agricultural office in the municipality reported that farmers have found ticks on their dogs which had frequented the same rough grazing area as used by the sheep in the farm, and in infields of other farms located at similar elevations (~850 masl); one tick from a dog was identified as *I. ricinus.*

| **District** | **Timespan** | **Farm id** | **Testprevalence** | **Meters above sea level- Farm** | **Meters above sea level - Pasture, lower bounds** | **Meters above sea level-pasture, higher bounds** |
| --- | --- | --- | --- | --- | --- | --- |
| **INLAND** | 1 | 20 | 0.33 | 208 | 300 | 600 |
| **INLAND** | 1 | 8* | 0.23 | 313 | 700 | 930 |
| **INLAND** | 1 | 6 | 0.32 | 314 | 880 | 1000 |
| **INLAND** | 1 | 9 | 0.23 | 340 | 869 | 1160 |
| **INLAND** | 1 | 16 | 0.47 | 396 | 479 | 900 |
| **INLAND** | 1 | 5* | 0.58 | 434 | 648 | 1148 |
| **INLAND** | 3 | 4 | 0.28 | 81 | 1100 | 1530 |
| **INLAND** | 3 | 21 | 0.17 | 215 | 500 | 750 |
| **INLAND** | 3 | 18 | 0.24 | 221 | 700 | 1000 |
| **INLAND** | 3 | 19 | 0.80 | 265 | 600 | 800 |
| **INLAND** | 3 | 15 | 0.31 | 295 | 757 | 900 |
| **INLAND** | 3 | 12 | 0.07 | 299 | 800 | 1000 |
| **INLAND** | 3 | 13 | 0.27 | 312 | 800 | 977 |
| **INLAND** | 3 | 7 | 0.07 | 376 | 752 | 1163 |
| **INLAND** | 3 | 10 | 0.40 | 398 | 600 | 855 |
| **INLAND** | 3 | 11 | 0.36 | 418 | 900 | 952 |
| **INLAND** | 3 | 5* | 0.45 | 434 | 648 | 1148 |
| **INLAND** | 3 | 14 | 0.20 | 436 | 755 | 1088 |
| **INLAND** | 3 | 2 | 0.40 | 440 | 400 | 600 |
| **INLAND** | 3 | 3 | 0.07 | 484 | 750 | 950 |
| **INLAND** | 3 | 1 | 0.33 | 492 | 1100 | 1520 |
| **INLAND** | 3 | 17 | 0.04 | 748** | 1000 | 1400 |
| **COAST** | 1 | 64 | 0.42 | 10 | 10 | 10 |
| **COAST** | 1 | 25 | 1.00 | 10 | 10 | 10 |
| **COAST** | 1 | 40 | 0.51 | 16 | 16 | 16 |
| **COAST** | 1 | 53* | 0.07 | 54 | 800 | 1000 |
| **COAST** | 1 | 54 | 0.33 | 54 | 100 | 200 |
| **COAST** | 1 | 31 | 0.53 | 63 | 200 | 850 |
| **COAST** | 1 | 32 | 0.75 | 71 | 600 | 800 |
| **COAST** | 1 | 61 | 0.80 | 85 | 100 | 200 |
| **COAST** | 1 | 56* | 0.04 | 125 | 250 | 400 |
| **COAST** | 1 | 29 | 0.43 | 199 | 199 | 199 |
| **COAST** | 1 | 60 | 0.36 | 210 | 500 | 800 |
| **COAST** | 1 | 57 | 0.83 | 304 | 304 | 304 |
| **COAST** | 2 | 24 | 1.00 | 14 | 14 | 14 |
| **COAST** | 2 | 38 | 0.89 | 45 | 45 | 45 |
| **COAST** | 2 | 45 | 0.02 | 53 | 600 | 900 |
| **COAST** | 2 | 22 | 1.00 | 56 | 56 | 56 |
| **COAST** | 2 | 27 | 0.90 | 63 | 63 | 63 |
| **COAST** | 2 | 30 | 0.75 | 63 | 400 | 650 |
| **COAST** | 2 | 23 | 1.00 | 74 | 74 | 74 |
| **COAST** | 2 | 36 | 0.73 | 77 | 77 | 77 |
| **COAST** | 2 | 37 | 0.00 | 96 | 96 | 96 |
| **COAST** | 2 | 41 | 0.00 | 136 | 136 | 136 |
| **COAST** | 2 | 26 | 1.00 | 139 | 139 | 139 |
| **COAST** | 2 | 33 | 0.00 | 139 | 139 | 139 |
| **COAST** | 2 | 42 | 0.00 | 161 | 161 | 161 |
| **COAST** | 2 | 58 | 0.07 | 174 | 174 | 174 |
| **COAST** | 2 | 49 | 0.00 | 181 | 181 | 181 |
| **COAST** | 2 | 63 | 0.23 | 182 | 182 | 182 |
| **COAST** | 2 | 28 | 0.00 | 201 | 201 | 201 |
| **COAST** | 2 | 34 | 0.20 | 203 | 203 | 203 |
| **COAST** | 2 | 55 | 0.03 | 249 | 250 | 400 |
| **COAST** | 2 | 62 | 0.16 | 302 | 400 | 650 |
| **COAST** | 3 | 35 | 0.57 | 30 | 400 | 600 |
| **COAST** | 3 | 52 | 0.45 | 33 | 200 | 850 |
| **COAST** | 3 | 46 | 0.68 | 75 | 75 | 75 |
| **COAST** | 3 | 39 | 1.00 | 81 | 81 | 81 |
| **COAST** | 3 | 59 | 0.52 | 86 | 130 | 200 |
| **COAST** | 3 | 48 | 0.97 | 89 | 89 | 89 |
| **COAST** | 3 | 43 | 0.09 | 97 | 97 | 97 |
| **COAST** | 3 | 47 | 1.00 | 99 | 200 | 850 |
| **COAST** | 3 | 51 | 1.00 | 115 | 150 | 300 |
| **COAST** | 3 | 56* | 0.52 | 125 | 250 | 400 |
| **COAST** | 3 | 44 | 0.17 | 156 | 250 | 450 |
| **COAST** | 3 | 50 | 0.58 | 180 | 400 | 700 |
| **FJORD** | 1 | 84 | 1.00 | 12 | 600 | 700 |
| **FJORD** | 1 | 66 | 0.27 | 41 | 41 | 41 |
| **FJORD** | 1 | 65 | 0.10 | 49 | 1400 | 1400 |
| **FJORD** | 1 | 67 | 1.00 | 55 | 480 | 700 |
| **FJORD** | 1 | 83 | 0.98 | 65 | 0 | 600 |
| **FJORD** | 1 | 68 | 1.00 | 78 | 480 | 700 |
| **FJORD** | 2 | 69 | 0.79 | 12 | 800 | 800 |
| **FJORD** | 2 | 77 | 0.87 | 43 | 500 | 700 |
| **FJORD** | 2 | 73 | 0.58 | 45 | 400 | 400 |
| **FJORD** | 2 | 89 | 0.79 | 57 | 800 | 1300 |
| **FJORD** | 2 | 82 | 1.00 | 77 | 77 | 77 |
| **FJORD** | 2 | 72 | 1.00 | 79 | 79 | 79 |
| **FJORD** | 2 | 70 | 1.00 | 163 | 1150 | 1300 |
| **FJORD** | 2 | 90 | 0.43 | 192 | 900 | 900 |
| **FJORD** | 2 | 71 | 0.36 | 241 | 241 | 241 |
| **FJORD** | 3 | 75 | 0.97 | 11 | 600 | 700 |
| **FJORD** | 3 | 76 | 0.80 | 13 | 500 | 900 |
| **FJORD** | 3 | 79 | 1.00 | 15 | 400 | 500 |
| **FJORD** | 3 | 85 | 1.00 | 16 | 16 | 16 |
| **FJORD** | 3 | 78 | 1.00 | 22 | 550 | 650 |
| **FJORD** | 3 | 80 | 1.00 | 23 | 23 | 23 |
| **FJORD** | 3 | 87 | 0.87 | 66 | 380 | 1000 |
| **FJORD** | 3 | 81 | 0.96 | 79 | 550 | 730 |
| **FJORD** | 3 | 74 | 0.80 | 81 | 500 | 900 |
| **FJORD** | 3 | 86 | 0.96 | 84 | 84 | 84 |
| **FJORD** | 3 | 88 | 1.00 | 93 | 500 | 900 |

**Table S2:** Distribution of livestock farms and human populations and the level of agricultural activity represented by hours spent on agricultural work in various municipalities in the 3 study areas during timespan 1(1980-1989), timespan 2(1990-1999) and timespan 3(2000-2008).

| **Location** | | **Livestock farms (N)** | | | **Human population (N)** | | | **Agriculture work (hours)** | | |
| --- | --- | --- | --- | --- | --- | --- | --- | --- | --- | --- |
| **District** | **Municipality** | **Timespan1** | **Timespan2** | **Timespan3** | **Timespan1** | **Timespan2** | **Timespan3** | **Timespan1** | **Timespan2** | **Timespan3** |
| INLAND | Kviteseid | 119 | 78 | 55 | 2975 | 2780 | 2617 | 279151 | 155469 | 90891 |
| INLAND | Fyresdal | 70 | 58 | 43 | 1438 | 1372 | 1350 | 170157 | 69261 | 33499 |
| INLAND | Tokke | 141 | 107 | 83 | 2757 | 2604 | 2452 | 278707 | 213712 | 113269 |
| INLAND | Vinje | 248 | 191 | 133 | 3970 | 3970 | 3766 | 553851 | 333642 | 189001 |
| INLAND | Bygland | 93 | 72 | 47 | 1512 | 1365 | 1306 | 241750 | 132264 | 66014 |
| COAST | Sandnes | 571 | 484 | 385 | 39631 | 48062 | 57033 | 1675222 | 1496485 | 926529 |
| COAST | Lund | 192 | 162 | 122 | 3060 | 3080 | 3111 | 410503 | 388222 | 220358 |
| COAST | Bjerkreim | 247 | 234 | 220 | 2222 | 2420 | 2481 | 828450 | 794676 | 625778 |
| COAST | Hå | 654 | 603 | 532 | 12636 | 13270 | 14589 | 2153668 | 2117462 | 1591263 |
| COAST | Time | 384 | 351 | 315 | 10879 | 12549 | 14252 | 1343000 | 1195860 | 915297 |
| COAST | Gjesdal | 217 | 197 | 181 | 6186 | 8031 | 9272 | 669041 | 586431 | 463301 |
| COAST | Kvitsøy | 24 | 21 | 17 | 527 | 502 | 522 | 70337 | 48126 | 23765 |
| FJORD | Vindafjord | 449 | 402 | 332 | 7933 | 8092 | 8133 | 1841696 | 1530267 | 1025189 |
| FJORD | Etne | 296 | 241 | 219 | 4052 | 3966 | 3910 | 732312 | 573482 | 399300 |

**Table S3**: Description of the climatic and environmental variables at infield/farm level and at rough pasture level that was significant after univariate testing.

| **INFIELD/FARM LEVEL** | |
| --- | --- |
| **Variable** | **Definition** |
| Moose | The number of bagged moose in the municipality where the farm reside |
| Red Deer | The number of bagged red deer in the municipality where the farm reside |
| Roe Deer | The number of bagged roe deer in the municipality where the farm reside |
| Sheep | The number of sheep in the municipality where the farm reside |
| NuFarms | The number of farms in the municipality where the farm reside |
| F_masl | The meters above sea level at which the farm is situated |
| Humans | The number of inhabitants in the municipality where the farm reside |
| Area | Denoting district 1,2 and 3 (INLAND,COAST and FJORD) |
| Timespan | Denoting the 3 decades; timespan 1(80s),timespan 2(90s) and timespan 3(00s) |
| Nu_patch | Number of patches of bush enroachment in a 500-m radius |
| Meanarea_p | Mean area of patches of bush enroachment intersected by a 500-m radius (m²) |
| Area_shrubi | Total area covered by patches of bush enroachment intersected by a 500-m radius (m²) |
| SatDefMean_May-Aug_ | Mean saturation deficit in May-August |
| RHMean_Oct-Mar_ | Mean relative humidity October-March |
| SatDefMean_May-Aug_ | Mean saturation deficit in May-August |
| TMean_Jan-Dec_ | Annual mean air temperature |
| TMeanSD_Apr_ | Daily mean air temperature standard deviation in april |
| SnoStartDays | Number of days from 1 September to snow depth ≥2 cm |
| SnoDepth_1-20_Days | Number of days from 1 September with snow depth of 1-20 cm |
| RRSum_May_ | Mean monthly precipitation in May |
| TDecr_÷5<_Days_Nov-Feb_ | Number of days between 1 Nov and 28 Feb where temperature decrease in GST from a day to the next day are >5 °C. |
| TDecr_÷5<_Days_Jan-Dec_ | Number of days pr year where temperature decrease in GST from a day to the next day are >5 °C. |
| TIncr_÷5<_Days_Nov-Feb_ | Number of days between 1 Nov and 31 Dec where temperature increase in GST from a day to the next day are >5 °C. |
| TIncr_+5<_Days_Jun_ | Number of days in June where temperature increase in GST from a day to the next day are >5 °C. |
| TIncr_+5<_Days_Nov_ | Number of days in November where temperature increase in GST from a day to the next day are >5 °C. |
| GSTmin_Jan-Dec_ | Annual mean of lowest daily Ground Surface Temperature (GST); monthly basis |
| GSTmax_Jan-Dec_ | Annual mean of highest daily Ground Surface Temperature (GST); monthly basis |
| GrowSeasDays | The length of the growing season. |
| **PASTURE LEVEL (ROUGH GRAZING)** | |
| **Variable** | **Definition** |
| Nu_patch | Number of patches of bush enroachment in a 500-m radius |
| Meanarea_p | Mean area of patches of bush enroachment intersected by a 500-m radius (m²) |
| Area_shrubi | Total area covered by patches of bush enroachment intersected by a 500-m radius (m²) |
| TMean_Jan-Dec_ | Annual mean air temperature |
| TMeanSD_Jan-Dec_ | Daily mean air temperature standard deviation; annual mean |
| SnoStartDays | Number of days from 1 September to snow depth ≥2 cm |
| SnoDepth_1-20_Days | Number of days from 1 September with snow depth of 1-20 cm |
| RRSum_Mar_ | Mean monthly precipitation in March |
| TDecr_÷5<_Days_Jan-Dec_ | Number of days pr year where temperature decrease in GST from a day to the next day are >5 °C. |
| TIncr_+5<_Days_May_ | Number of days in May where temperature increase in GST from a day to the next day are >5 °C. |
| TIncr_+5<_Days_Oct_ | Number of days in Oct where temperature increase in GST from a day to the next day are >5 °C. |
| GSTmin_Jan-Dec_ | Annual mean of lowest daily Ground Surface Temperature (GST) |
| GSTmax_Jan-Dec_ | Annual mean of highest daily Ground Surface Temperature (GST) |
| SnoSS | Consist of SnoEndDays, SnoSum, SnoDepth≥2Days and SnoDepth>20Days |
| Snomean | Taking the mean of all the snowvariables |
| BlackFrdays | Number of days from 1 September with Black frost; daily GST < 0°C and ground bare of snow or snow depth < 2 cm. |
| FTDays-SnoDepth_<2_ FTDays-SnoDepth_≥2_ | Mean of FTDays-SnoDepth_<2_ and FTDays-SnoDepth_≥2_ |
| GrowSeasDays | The length of the growing season. |

**Table S4**: Descriptive statistics of the explanatory variables and their univariate relationship with the outcome variable (presence of antibodies to *A. phagocytophilum* in sheep serum).

| Variable name | Descriptive values | | Categorized variables* | Prevalence (Farm level) | |
| --- | --- | --- | --- | --- | --- |
|  | Mean | 90% range | Levels | Mean | 50% range |
| Meanarea_p | 2109 | 0 - 7975 | 0: | 0.55 | 0.25 – 0.91 |
|  |  |  | 0 – 900: | 0.45 | 0.19 – 0.78 |
|  |  |  | 900 – 2268: | 0.68 | 0.27 – 1 |
|  |  |  | 2268 – 23767: | 0.42 | 0.20 – 0.46 |
| Area_shrubi | 0:  1:  2:  3: | 22  19  24  22 | 0 | 0.47 | 0.09 - 0.87 |
|  |  |  | 1 | 0.42 | 0.22 – 0.45 |
|  |  |  | 2 | 0.63 | 0.31 – 0.97 |
|  |  |  | 3 | 0.61 | 0.36 – 0.94 |
| Pasture | Farm  Mountain | 31  56 | Farm | 0.59 | 0.14 – 1 |
|  |  |  | Mountain | 0.51 | 0.26 – 0.80 |
| BlackFrdays | - 0.08 | -0.92 – 1.00 | -0.92 | 0.57 | 0.18 – 1 |
|  |  |  | -0.92 – -0.20 | 0.64 | 0.34 – 1 |
|  |  |  | -0.20 – 0.63 | 0.40 | 0.23 – 0.53 |
|  |  |  | 0.63 – 6.32 | 0.59 | 0.32 – 0.85 |
| RedDeer | 0.08 | 0.00 – 0.40 | 0.00 – 0.00 | 0.39 | 0.14 – 0.52 |
|  |  |  | 0.00 – 0.005 | 0.45 | 0.17 – 0.68 |
|  |  |  | 0.005 – 0.131 | 0.54 | 0.20 – 1 |
|  |  |  | 0.131 – 0.402 | 0.83 | 0.80 – 1 |
| NuFarms | 0.59 | 0.03 – 2.07 | 0.03 – 0.21 | 0.29 | 0.18 – 0.39 |
|  |  |  | 0.21 – 0.36 | 0.76 | 0.75 – 1 |
|  |  |  | 0.36 – 0.58 | 0.48 | 0.02 – 0.75 |
|  |  |  | 0.58 – 2.13 | 0.63 | 0.34 – 1 |
| TDecr_÷5<_Days_Jan-Dec_ | 0.01 | 0.00 – 0.03 | 0.00 – 0.004 | 0.48 | 0.21 – 0.79 |
|  |  |  | 0.004 – 0.007 | 0.76 | 0.52 – 1 |
|  |  |  | 0.007 – 0.013 | 0.45 | 0.14 – 0.79 |
|  |  |  | 0.013 – 0.038 | 0.47 | 0.25 – 0.62 |
| RRSum_May_ | 91 | 61 - 120 | 58 – 75 | 0.40 | 0.02 – 0.78 |
|  |  |  | 75 – 87 | 0.49 | 0.26 – 0.76 |
|  |  |  | 87 – 108 | 0.49 | 0.24 – 0.79 |
|  |  |  | 108 – 147 | 0.78 | 0.63 – 0.99 |
| TMeanSD_April_ | 2.36 | 1.81 – 2.70 | 1.73 – 2.30 | 0.59 | 0.34 – 0.90 |
|  |  |  | 2.30 – 2.38 | 0.77 | 0.60 – 1 |
|  |  |  | 2.38 – 2.47 | 0.37 | 0.17 – 0.51 |
|  |  |  | 2.47 – 2.79 | 0.39 | 0.02 – 0.85 |
| TIncr_+5<_Days_Jun_ | 0.05 | 0.00 – 0.19 | 0.00 | 0.69 | 0.40 – 1 |
|  |  |  | 0.00 – 0.04 | 0.59 | 0.39 - 0.82 |
|  |  |  | 0.04 – 0.05 | 0.59 | 0.31 – 0.95 |
|  |  |  | 0.05 – 0.57 | 0.20 | 0.02 – 0.28 |
| RRSum_Mar__ | 114 | 0 - 333 | 0.00 | 0.57 | 0.18 – 1 |
|  |  |  | 0 – 97 | 0.34 | 0.20 – 0.40 |
|  |  |  | 97 – 211 | 0.34 | 0.23 – 0.45 |
|  |  |  | 211 – 451 | 0.72 | 0.57 – 0.99 |
| SnoStartDays | 93.5 | 65.9 – 121.6 | 60.3 – 77.4 | 0.25 | 0.07 – 0.35 |
|  |  |  | 77.4 – 95.0 | 0.53 | 0.24 – 0.89 |
|  |  |  | 95.0 – 107.0 | 0.71 | 0.44 – 1 |
|  |  |  | 107.0 – 124.7 | 0.69 | 0.51 – 1 |
| RHMean_Oct-Mar_ | 88.3 | 85.1 – 90.9 | 80.3 – 86.4 | 0.78 | 0.55 – 1 |
|  |  |  | 86.4 – 89.2 | 0.51 | 0.09 – 0.91 |
|  |  |  | 89.2 – 89.8 | 0.44 | 0.17 – 0.75 |
|  |  |  | 89.8 – 91.6 | 0.38 | 0.21 – 0.50 |
| RoeDeer | 0.07 | 0.00 – 0.23 | 0.00 – 0.02 | 0.59 | 0.35 – 0.95 |
|  |  |  | 0.02 – 0.05 | 0.27 | 0.07 – 0.36 |
|  |  |  | 0.05 – 0.06 | 0.81 | 0.74 – 1 |
|  |  |  | 0.06 – 0.26 | 0.57 | 0.31 – 1 |
| Moose | 0.05 | 0.00 – 0.22 | 0.0000 – 0.0004 | 0.47 | 0.06 – 0.85 |
|  |  |  | 0.0004 – 0.0033 | 0.62 | 0.43 – 0.97 |
|  |  |  | 0.003 – 0.038 | 0.83 | 0.80 – 1 |
|  |  |  | 0.038 – 0.337 | 0.27 | 0.17 – 0.35 |
| GrowSeasDays | 284 | 160 – 347 | 133 – 233 | 0.32 | 0.19 – 0.40 |
|  |  |  | 233 – 298 | 0.47 | 0.20 – 0.80 |
|  |  |  | 298 – 334 | 0.56 | 0.16 – 0.94 |
|  |  |  | 334 – 349 | 0.82 | 0.72 – 1.00 |

*Continuous variables where categorized in 4 equal parts (based on quantiles)**Additional file 1 References**

1. Jore S, Viljugrein H, Hofshagen M, Brun-Hansen H, Kristoffersen A, Nygard K, Brun E, Ottesen P, Saevik B, Ytrehus B. 2011 Multi-source analysis reveals latitudinal and altitudinal shifts in range of Ixodes Ricinus at its northern distribution limit. *Parasites & Vectors* **4,** 84.

2. Mehl R. 1983 The distribution and host relations of Norwegian ticks (Acari, Ixodides). *Fauna norvegica Series B* **30,** 46-51.

3. Tambs-Lyche H. 1943 Ixodes ricinus og piroplasmosen i Norge (Meddelelse fra Bergens Museums zoologiske avdeling). *Norsk veterinærtidsskrift* **55,** 337-542.

4. Woldehiwet Z, Horrocks BK. 2005 Antigenicity of ovine strains of Anaplasma phagocytophilum grown in tick cells and ovine granulocytes. *J Comp Pathol.* **132,** 322-328. (S0021-9975(05)00006-X [pii];10.1016/j.jcpa.2004.12.002 [doi])

5. Woldehiwet Z, Yavari C. 2012 Evaluation of an indirect enzyme-linked immunosorbent assay (ELISA) for the detection of antibodies against Anaplasma phagocytophilum in sheep. *J Comp Pathol.* **146,** 116-121. (S0021-9975(11)00066-1 [pii];10.1016/j.jcpa.2011.04.004 [doi])

6. A. Jansson, O. E. Tveito, P. Pirinen, and M. Scharling. NORGRID - a preliminary investigation on the potential for creation of a joint Nordic gridded climate dataset. 03/2007. 2007. Met.no, Report 03/2007.

7. M. Mohr. Comparison of version 1.1 and 1.0 of gridded temperature and precipitation data for Norway. 2009. Met.no. Note 19/2009.

8. Tveito OE, Bjordal I, Skjelvag AO, Aune B. 2005 A GIS-based agro-ecological decision system based on gridded climatology. *Meteorological Applications* **12,** 57-68.

9. O. E. Tveito, E. J. Førland, R. Heino, I. Hanssen-Bauer, H. Alexandersson, B. Dahlström, A. Drebs, C. Kern-Hansen, T. Jónsson, E. Vaarby Laursen, and Y. Westman. Nordic temperature maps. 09/00. 2000. DNMI. ISSN 0805-9918.

10. E. J. Førland. Lokalklima på vestlandskysten (Local climate in western Norway, in Norwegian with English summary).Klima 1984, 6,24-36.

11. E. J. Førland. Nedbørens høydeavhengighet (Precipitation and topography, in Norwegian with English summary). Klima 1979, 1,3-24.

12. R. V. Engeset, O. E. Tveito, E. Alfnes, Z. Mengistu, H.-C. Udnæs, K. Isaksen, and E. J. Førland. Snow map system for Norway. Proceedings XXIII Nordic Hydrological Conference 2004, 8-12 August 2004, Tallin, Estonia. 2012.

13. S. Alfnes. Snow depth algorithms - compaction of snow. 2008. NVE internal report.

14. M. Reistad, Ø. Breivik, and H. Haakenstad. A High-resolution Hindcast Study for the North Sea, the Norwegian Sea and the Barents Sea, in Proceedings of the 10th International Workshop on Wave Hindcasting and Forecasting and Coastal Hazard Symposium. 2007.

15. Reistad M, Breivik O, Haakenstad H, Aarnes OJ, Furevik BR, Bidlot JR. 2011 A high-resolution hindcast of wind and waves for the North Sea, the Norwegian Sea, and the Barents Sea. *Journal of Geophysical Research-Oceans* **116,**

16. P. Undén, L. Rontu, H. Järvinen, P. Lynch, J. Calvo, G. Cats, J. Cuaxart, K. Eerola, C. Fortelius, J. A. Garcia-Moya, C. Jones, G. Lenderlink, A. McDonald, R. McGrath, B. Navascues, N. W. Nielsen, V. Ødegaard, E. Rodriguez, M. Rummukainen, R. Rööm, K. Sattler, B. H. Sass, H. Savijärvi, B. W. Schreur, R. Sigg, H. The, and A. Tijm. HIRLAM-5 Scientific Documantation,HIRLAM-5 Project. 2002. SMHI, S-601767 Norrköping, Sweden.

17. Westermann S, Boike J, Langer M, Schuler TV, Etzelmuller B. 2011 Modeling the impact of wintertime rain events on the thermal regime of permafrost. *Cryosphere* **5,** 945-959.

18. Dall'Amico M, Endrizzi S, Gruber S, Rigon R. 2011 A robust and energy-conserving model of freezing variably-saturated soil. *Cryosphere* **5,** 469-484.

19. Tucker CJ, Sellers PJ. 1986 Satellite Remote Sensing of Primary Production. *Int J. Remote Sensing* **7,** 1395-1416.

20. Prince SD. 1991 Satellite remote sensing for primary production: Comparison of results for Sahelian grassland 1981-1988. *Journal of Remote Sensing*

21. Sellers PJ. 1985 Canopy Reflectance, Photosynthesis and Transpiration. *Int J. Remote Sensing* **6,** 1335.

22. Myneni RB, Hall FG, Sellers PJ, Marshak AL. 1995 The Interpretation of Spectral Vegetation Indexes. *Ieee Transactions on Geoscience and Remote Sensing* **33,** 481-486.

23. Slayback DA, Pinzon JE, Los SO, Tucker CJ. 2003 Northern hemisphere photosynthetic trends 1982-99. *Global Change Biology* **9,** 1-15.

24. Bown KJ, Lambin X, Telford GR, Ogden NH, Telfer S, Woldehiwet Z, Birtles RJ (2008) Relative importance of Ixodes ricinus and Ixodes trianguliceps as vectors for Anaplasma phagocytophilum and Babesia microti in field vole (Microtus agrestis) populations. *Applied and Environmental Microbiology,* **74,** 7118-7125.

25. Dreher UM, de la Fuente J, Hofmann-Lehmann R, et al (2005) Serologic cross-reactivity between Anaplasma marginale and Anaplasma phagocytophilum. *Clinical and Diagnostic Laboratory Immunology,* **12,** 1177-1183.

26. Dyrrdal AV, Isaksen K, Hygen HO, Meyer NK, in press. 2013 Past changes in frequency, intensity and spatial occurrence of meteorological triggering variables relevant for natural hazards in Norway. in press in *Climate Research*

27. I. Hanssen-Bauer, H. Drange, E. J. Førland, L. A. Roald, K. Y. Børsheim, H. Hisdal, D. Lawrence, A. Nesje, S. Sandven, A. Sorteberg, S. Sundby, K. Vasskog, and B. Ådlandsvik. Climate in Norway: Background material to the NOU Climate Adaption. 2009. Norsk klimasenter,Oslo. NOU.
